# Alignment-free comparative genomic screen for structured RNAs using coarse-grained secondary structure dot plots

**DOI:** 10.1186/s12864-017-4309-y

**Published:** 2017-12-02

**Authors:** Yuki Kato, Jan Gorodkin, Jakob Hull Havgaard

**Affiliations:** 1 Department of RNA Biology and Neuroscience, Graduate School of Medicine, Osaka University, 2-2 Yamadaoka, Suita, 565-0871 Japan; 2 Center for non-coding RNA in Technology and Health (RTH), University of Copenhagen, Groennegaardsvej 3, Frederiksberg, 1870 Denmark

**Keywords:** Non-coding RNA, Secondary structure, Gene finding

## Abstract

**Background:**

Structured non-coding RNAs play many different roles in the cells, but the annotation of these RNAs is lacking even within the human genome. The currently available computational tools are either too computationally heavy for use in full genomic screens or rely on pre-aligned sequences.

**Methods:**

Here we present a fast and efficient method, DotcodeR, for detecting structurally similar RNAs in genomic sequences by comparing their corresponding coarse-grained secondary structure dot plots at string level. This allows us to perform an all-against-all scan of all window pairs from two genomes without alignment.

**Results:**

Our computational experiments with simulated data and real chromosomes demonstrate that the presented method has good sensitivity.

**Conclusions:**

DotcodeR can be useful as a pre-filter in a genomic comparative scan for structured RNAs.

**Electronic supplementary material:**

The online version of this article (doi:10.1186/s12864-017-4309-y) contains supplementary material, which is available to authorized users.

## Background

Non-coding RNAs (ncRNAs), which are RNAs not translated into proteins, have many different functions within the cells. In the current version of the human genome (Ensembl 86.38, June 2016), there are 22,219 non-coding genes and 20,441 protein coding genes annotated, indicating that ncRNAs are of great importance. While RNA-sequencing (RNA-seq) is routinely used to locate ncRNA transcripts [1], computational methods for detecting ncRNAs are needed since some ncRNAs are so lowly expressed that they can be hard if not impossible to detect in RNA-seq data without prior knowledge. Furthermore, not all ncRNAs are expressed in all cell types, which also complicates detection of novel ncRNAs with experimental methods. A significant part of the ncRNAs have a secondary structure, which is conserved between species. This structural conservation can be used to computationally locate ncRNA genes in the genomes [2].

Comparative methods, which in this case focus on structural conservation, are significantly more accurate than methods based on single sequences. Comparative structure prediction can be classified into three groups, namely: align-then-fold, fold-then-align and simultaneous align-and-fold approaches [3]. The most accurate methodology is the last one, and its pioneering work based on dynamic programming is presented by Sankoff [4]. This algorithm, however, requires too high computational cost in terms of run-time and memory usage for any practical use. To circumvent this problem, restricted, approximated or alternative versions of the Sankoff-style algorithm, including FOLDALIGN [5–8], Dynalign [9–11], CMfinder [12], LocARNA [13, 14], Murlet [15], RAF [16] and DAFS [17], have been published with/without application to ncRNA discovery. However, these methods are still too slow to perform an all-against-all scan of all windows from genomic sequences.

Examples of the align-then-fold algorithms are RNAz [18], evofold [19] and PETfold [20]. While these methods are fast and deliver accurate results, they also require that the input sequences have been aligned correctly. Because these methods rely on sequence alignment, they are limited to sequences with high sequence similarity. The impact of sequence similarity on structural alignment has been discussed by Torarinsson et al. [21] and Gardner et al. [22].

To predict whether two structures are similar, the distance between the two RNA secondary structures can be calculated. This can be done by calculating the tree edit distance between the dot bracket representation of the RNA secondary structures as in RNAdistance [23, 24], or by calculating the distance between two vectors based on the base-pairing probability dot plots as done by RNApdist [23, 24]. A relaxed base pair score [25], which is a generalized base pair metric, has also been proposed to gain a more biologically realistic distance. From another viewpoint of image analysis, a few studies have been proposed where the distance is derived from a combination of histogram correlations and a geometrical distance measure [26–28].

GraphClust [29] clusters structured RNA sequences based on hashing of the RNA structures, followed by refinement of the clusters using structural alignment. RNAsscClust [30] extends GraphClust to include information from multiple sequences when multiple alignments of RNAs are used as input. Structator [31] uses affix arrays to map known RNA structures to the genome very fast.

Unfortunately, most of the methods stated above either focus on the folding of known RNA genes and not on finding RNA structures in long genomic sequences, require correctly aligned input sequences, or have high computational costs. Thus, there is a lack of tools that can be used to quickly predict structured RNAs in ‘unalignable’ genomic sequences.

In this work, an alignment-free approach to the comparative genomic screen for structured ncRNAs is presented, which can be used as a first step of a complete pipeline for the *de novo* prediction of structured RNAs. The algorithm is intended to be a pre-filter that significantly reduces the input space without removing the structured RNAs. The output of the method can then be used as input for more precise, but slower methods.

The computational method DotcodeR, DOT plot enCODEr for RNA is presented in this paper. It uses structural similarity to search for RNA structures in genomic sequences. DotcodeR applies a sliding window framework and employs coarse-grained secondary structure dot plots to compare two potential structured RNA regions. The coarse-grained secondary structure dot plots are transformed into binary vectors, and the similarity of two vectors is calculated as a simple dot product. Due to speed of the dot product calculation, the all-against-all comparison of all window pairs from two chromosomes is feasible even without anchoring the windows on alignable sequences. To test the method, a search for ncRNAs conserved between human chromosome 21 and mouse chromosome 19 was conducted, and the results indicate that DotcodeR can be used to locate known structured RNAs while reducing the search space by 97.1%. This shows that the method is suitable as a pre-filter in genomic screen.

## Methods

### DotcodeR overview

**Fig. 1:**
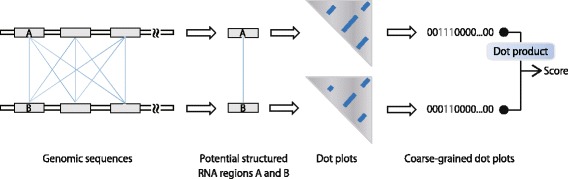
A schematic diagram of DotcodeR. Potential structured RNA genes are assumed to be taken from the sliding windows in two genomic sequences, which are shown as gray rectangles. In this figure the windows are drawn to be non-overlapping but in reality adjacent windows overlap. All windows from one sequence are compared to all windows from the other sequence as indicated by the thin blue lines. For these sequence windows, the secondary structure dot plots are computed using the partition function-based method. Coarse-grained dot plots (binary vectors) are then obtained by the conversion rule stated in the main text. Finally, a structural similarity score can be calculated by dot product between two binary vectors

The purpose of the method DotcodeR is to locate structured RNAs conserved between two genomic sequences. To this end, subsequences are extracted from the genomic sequences, and these subsequences are compared to each other by calculating the dot product of binary vectors that reflect local ensemble secondary structures. The score is used to predict whether the subsequences have a similar RNA structure or not (see Fig. 1).

### Secondary structure dot plot

The binary vectors used in the dot product are based on the base-pairing probabilities. This section describes how the base-pairing probabilities are calculated.

Let *x*=*x*_1_ *x*_2_…*x*_*L*_ be an RNA sequence, where *x*_*i*_∈{A,C,G,U} for 1≤*i*<*j*≤*L*. The RNA sequence *x* can fold into a secondary structure *y*, which consists of canonical base pairs such as A-U, G-C and G-U. The posterior probability *p*_*ij*_ that *x*_*i*_ is paired with *x*_*j*_ given the RNA sequence *x* can be calculated by

$$p_{ij} = \frac{1}{Z} \sum_{y \in \mathcal{S}_{ij}(x)} e^{-\frac{G(y)}{RT}}, $$

where $Z = \sum _{y \in \mathcal {S}(x)} e^{-\frac {G(y)}{RT}}$ is the partition function over the set $\mathcal {S}(x)$ of all secondary structures of *x*, $\mathcal {S}_{ij}(x)$ is the set of all secondary structures of *x* with *x*_*i*_ and *x*_*j*_ paired, *G*(*y*) is the free energy of *y*, *R* is the gas constant, and *T* is the temperature. In the actual case, *p*_*ij*_ is calculated by dynamic programming that employs additional partition functions including *Z*_*ij*_ defined over all secondary structures on a sequence interval [*i*,*j*] (see [32] for further details).

When addressing a genomic screen for structured RNAs with a sliding window of fixed size *w*, we need to know ‘local’ base-pairing probabilities within that window. A good solution is to consider the partition function $Z_{ij}^{u,w}$ over all secondary structures on the interval [*i*,*j*] with the window [*u*,*u*+*w*] folded. More specifically, $Z_{ij}^{u,w} = Z_{ij}$ if [*i*,*j*]⊆[*u*,*u*+*w*], and $Z_{ij}^{u,w} = 0$ otherwise. The local base-pairing probability $p_{ij}^{u,w}$ can also be calculated by dynamic programming using these partition functions (see [33] for details).

A secondary structure *dot plot* for an RNA sequence is a base-pairing probability matrix for that sequence, where each (*i*,*j*) element is *p*_*ij*_ (or $p_{ij}^{u,w}$). Note that we have only to consider upper triangular part of the matrix because of the relationship *i*<*j*. A dot plot for a given RNA sequence can be computed by the partition function-based approach stated above, which is implemented by RNAfold [23] for global base-pairing probabilities and RNAplfold [33] for local pairing probabilities in the ViennaRNA package [24]. In this work, we used RNAplfold to compute dot plots that correspond to local base-pairing probabilities.

### Binary vectors

The binary vector is constructed from a given dot plot using a sliding window of fixed size 2*d*+1. It should be noted that the window introduced here is different from the previous one of size *w* for genomic screen, and it is arranged in the diagonal-like way on the dot plot (see ‘Moving window’ in Fig. 2 as an example of *d*=1). It is also to be noted that the moving window has two shapes depending on the first position of the window center put on the diagonal (e.g. ‘L’ shape and its inverse for *d*=1 as shown in Fig. 2). Let *p*_*ij*_ denote a local base-pairing probability in the dot plot, which corresponds to the central position of the window, and *θ* be a threshold. Here we consider the following rule that converts pairing probabilities within the window into a binary digit *b*∈{0,1}:

1. if the next of cell (*i*,*j*) in the window is (*i*+1,*j*):
*b*=1 if $p_{ij} + \sum _{\delta =1}^{d} \left (p_{i-\lfloor \frac {\delta }{2}\rfloor,\ j-\lceil \frac {\delta }{2}\rceil } + p_{i+\lceil \frac {\delta }{2}\rceil,\ j+\lfloor \frac {\delta }{2}\rfloor } \right) > \theta $;

**Fig. 2:**
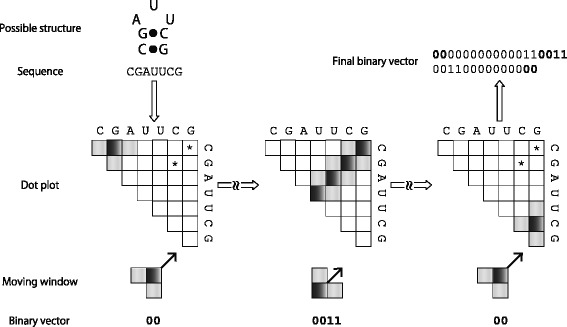
An illustration for explaining how to create a binary vector from an example RNA sequence. A moving window with 2*d*+1 cells (*d*=1 in this example) is shown in the ‘Moving window’ row. The range that the window can move along one anti-diagonal is shown by shaded cells in dot plots, and a black cell in the window corresponds to *p*_*ij*_ used in the main text. Note that an asterisk in the dot plot indicates a high probability of forming a base pair at the corresponding position, and the other cells without asterisk show zero probability. The window starts moving at the upper left corner on the diagonal and moves to the anti-diagonal way, which is indicated by the arrow shown in the ‘Moving window’ row. When all the cells of the window reaches the boundary of the dot plot, the window is moved back to the start position of this anti-diagonal scan and slidden by one lower right diagonal step. Note that at this moment the shape of the window is changed, e.g. the inverse ‘L’ shape into the ‘L’ shape. Repeat this scan until the window crosses over the lower right corner of the dot plot. Bold digits in the ‘Binary vector’ row are also embedded in the ‘Final binary vector,’ which are shown by bold digits in the calculation order
*b*=0 if $p_{ij} + \sum _{\delta =1}^{d} \left (p_{i-\lfloor \frac {\delta }{2}\rfloor,\ j-\lceil \frac {\delta }{2}\rceil } + p_{i+\lceil \frac {\delta }{2}\rceil,\ j+\lfloor \frac {\delta }{2}\rfloor } \right) \le \theta $;
2. else if the next of cell (*i*,*j*) in the window is (*i*,*j*+1):
*b*=1 if $p_{ij} + \sum _{\delta =1}^{d} \left (p_{i-\lceil \frac {\delta }{2}\rceil,\ j-\lfloor \frac {\delta }{2}\rfloor } + p_{i+\lfloor \frac {\delta }{2}\rfloor,\ j+\lceil \frac {\delta }{2}\rceil } \right) > \theta $;
*b*=0 if $p_{ij} + \sum _{\delta =1}^{d} \left (p_{i-\lceil \frac {\delta }{2}\rceil,\ j-\lfloor \frac {\delta }{2}\rfloor } + p_{i+\lfloor \frac {\delta }{2}\rfloor,\ j+\lceil \frac {\delta }{2}\rceil } \right) \le \theta $.

Note that in the above conditions, ‘next’ means a cell in the fixed window, and we set the parameters as *d*=1 and *θ*=0.1 in the subsequent computational experiments.

Based on this conversion rule, a coarse-grained dot plot can be defined as a binary vector obtained by performing the following pseudo code (see also Fig. 2):



For windows that do not contain exactly 2*d*+1 probabilities on the boundary of the dot plot, the summing operation is performed with probabilities available in that window.

### DotcodeR

Figure 1 illustrates how DotcodeR works when a pair of genomic sequences is given. Two potential structured RNA genes (subsequences) are picked up by the sliding window in the two genomic sequences. Note that the window is to be moved along the genomes by some step size *s*, which is smaller than the window size *w*. First, the binary vectors are calculated for all windows in the two genomes. Once these coarse-grained dot plots are computed, dot products are calculated in all-against-all comparison of the vectors from the two genomes to quantify the structural similarity of the subsequences.

The theoretical run-time of this genomic scan algorithm is evaluated as follows. We let *L* denote the length of the longer genomic sequence of the two. The first step that computes the local base-pairing probability matrices by RNAplfold, requires *O*(*L* *w* ^2^) time for each genome [33]. The second step needs $O\left (\frac {(L-w)^{2}}{s^{2}}\right)$ comparisons of windows between the two genomic sequences. The binary vectors are *O*(*w* ^2^) long, and hence it takes *O*(*w* ^2^) to calculate the dot product between two vectors. Therefore, the run-time of DotcodeR can be evaluated as $O\left (Lw^{2} + \frac {(L-w)^{2}w^{2}}{s^{2}}\right)$.

### Datasets

#### Benchmark data

To benchmark the method, a positive dataset consisting of known structured RNAs was created. It is based on sequences from the Rfam 12.0 database [34]. The sequences in the dataset were selected in the following way:

1. Remove sequences that have unpaired nucleotides in columns with at least 80% gaps;
2. Remove sequences that have more than 20% gaps;
3. Remove sequences from families with fewer than 20 members;
4. Redundancy is reduced to at most 90% identity within a family;
5. Randomly select five sequences from each clan.

The first two steps are aimed at removing outlier sequences in the alignments. The consensus structure of some families in Rfam contains very few base pairs. Many of the structures with a low number of base pairs belong to families with few member sequences (data not shown). Step 3 removes a large part of these lightly structured non-coding RNA families.

Sequences were split into two disjoint subsets for training and testing. The purpose of the training is to determine the optimal score cutoff as described in the next section. Sequences from the same clan/family are all either in the training or the test dataset to limit over-fitting. Rfam contains many families from the miRNAs. These families have the same structure to a large extent, and all miRNA sequences were therefore grouped into one family. This was also done for the snoRNAs for similar reasons.

The training set has 73 families and 347 sequences, whereas the test set contains 47 families and 210 sequences. Details of these RNA families can be found in Additional file 1: Tables S1 and S2.

Next, two negative datasets for each family were created as follows:

- The ‘gene-shuffled’ dataset consists of shuffled sequences from the positive dataset. This dataset has the same number of negative sequences as that of the positive dataset, and the GC-content is the same.
- The ‘genome-shuffled’ dataset consists of shuffled sequences taken randomly from human chromosome 22 (GRCh38 [35]). The sequences in this dataset have a GC-content similar to that of the chromosome rather than that of the positive dataset.

All these negative sequences were shuffled while preserving the dinucleotide distribution [36].

For each positive/negative sequence in the dataset, we next created the corresponding ‘simulated short genomic sequence’ by adding dinucleotide-shuffled sequences of the original RNA gene to both ends of that gene. These added sequences can be considered as flanking regions. In the tests, the sliding window must overlap with the gene regions.

#### Genomic sequences

To evaluate the performance of DotcodeR on real genomic sequences, GRCh38 human chromosome 21 and GRCm38 mouse chromosome 19 were used. The chromosomes as well as their gene annotations were taken from Ensembl [35]. The annotated snoRNAs were further subdivided into H/ACA box and C/D box snoRNAs, since these two classes have different structures. These chromosomes were selected because chromosome 21 is the smallest human chromosome, and mouse chromosome 19 is the mouse chromosome with the least amount of sequence similarity to human chromosome 21.

Before running DotcodeR, regions annotated as repeats were removed using the repeat-masking available from the UCSC Genome Browser [37]. Window pairs from regions covered by human–mouse chained BLASTZ alignments [38] from the UCSC Genome Browser were also removed because these regions can be investigated using alignment-based methods.

### Evaluation metrics

We evaluated the predictive performance by calculating sensitivity (SEN), specificity (SPC), positive predictive value (PPV), negative predictive value (NPV) and Matthews correlation coefficient (MCC), defined by

$$\begin{array}{@{}rcl@{}} \text{SEN} &=& \frac{\text{TP}}{\text{TP}+\text{FN}},\quad \text{SPC} = \frac{\text{TN}}{\text{TN}+\text{FP}},\\ \text{PPV} &=& \frac{\text{TP}}{\text{TP}+\text{FP}},\quad \text{NPV} = \frac{\text{TN}}{\text{TN}+\text{FN}},\\ \text{MCC} &=& \frac{\text{TP} \times \text{TN} - \text{FP} \times \text{FN}}{\sqrt{(\text{TP}+\text{FP})(\text{TP}+\text{FN})(\text{TN}+\text{FP})(\text{TN} +\text{FN})}}, \end{array} $$

where TP is the number of true positives, TN is the number of true negatives, FP is the number of false positives, and FN is the number of false negatives.

### Software

The program DotcodeR is implemented in C++ and available along with the benchmark data at https://github.com/ykat0/dotcoder/. To compute secondary structure dot plots, we used the ViennaRNA package 2.1.9.

## Results

### Benchmarking DotcodeR

A discrimination test was carried out on the benchmark data of simulated short genomes for each RNA family as defined in the previous section using DotcodeR along with competitive methods. More precisely, we investigated similarity scores calculated by the comparative methods to discriminate between real RNA genes and shuffled ones taken from the sliding windows. Note that here we have three types of window comparisons, i.e. real-against-real, real-against-shuffled and shuffled-against-shuffled ones, and the way of evaluating performance depends on the type of comparison. For window comparison between real subsequences only, prediction can be evaluated as ‘per-gene’ evaluation, where if some similarity score is larger than a cutoff in each pair of ‘input short genomes,’ this pair of genes can be evaluated as true positive (TP), otherwise false negative (FN). In contrast, for comparison including shuffled subsequences, we used ‘per-window’ evaluation, where if a score is larger than the cutoff in each pair of ‘windows,’ this pair of subsequences can be evaluated as false positive (FP), otherwise true negative (TN). With these measures, we can evaluate the predictive performance of each comparative method by drawing the receiver operating characteristic (ROC) curve, employing true positive rate and false positive rate.

**Fig. 3:**
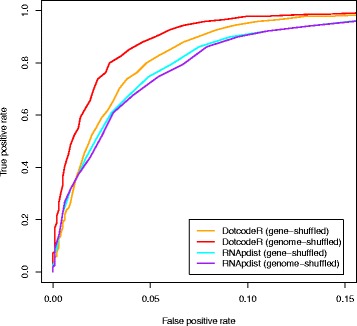
ROC curves showing predictive performance of the comparative methods on the test set of simulated short genomes. Comparison was carried out between DotcodeR and RNApdist. Here we used the window size of 120 nt and the step size of 30 nt. Note that accuracy was calculated by averaging over all results of the families in the dataset. The terms ‘gene-shuffled’ and ‘genome-shuffled’ refer to how the negative datasets were generated

As the proposed method is to be used to pre-screen for other methods, the sensitivity of DotcodeR has to be high. High sensitivity ensures high true positive rate, but unfortunately also leads to high false positive rate. When a tool is to be used as pre-screen, a high true positive rate is important as it keeps the real genes in the dataset. The false positive rate is less important since false positives will be removed by subsequent screens. The sensitivity of 90% on the training set was therefore selected, which resulted in a score cutoff of 20 (see Table 1 for performance on training and test datasets). ROC curves for DotcodeR used on the test data can be seen in Fig. 3 and on the training data in Additional file 1: Figure S5. A window size of 120 and a step size of 30 were used throughout all evaluations.

**Table 1:** Prediction accuracy of DotcodeR on each dataset of simulated short genomes

Dataset	Negative data	SEN	SPC	PPV	NPV	MCC
Training	Gene-shuffled	0.906	0.930	0.070	0.999	0.070
	Genome-shuffled	0.906	0.932	0.065	0.999	0.068
Test	Gene-shuffled	0.902	0.925	0.072	0.999	0.075
	Genome-shuffled	0.902	0.947	0.087	0.999	0.053

Here we used the window size of 120 nt, the step size of 30 nt and the cutoff of 20. Note that accuracy was calculated by averaging over all results of the families in each dataset

**Fig. 4:**
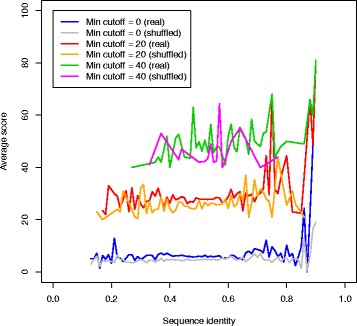
DotcodeR score as a function of sequence identity on the test set of simulated short genomes. The scores used in the *y*-axis are averaged over scores in all the families contained in the datasets. The min cutoff *c*_*min*_∈{0,20,40} means that we consider only scores of at least *c*_*min*_ to investigate the relationship

Sequence identity between the windows as well as the GC-content of the sequence might influence the performance of the method. Figure 4 (test dataset) and Additional file 1: Figure S11 (training dataset) show the dependency of the score as a function of sequence identity for different levels of score cutoffs. To calculate the scores on the shuffled sequences, the sequences from the positive set were aligned, and the alignments were shuffled preserving the dinucleotide content [39]. The figure shows that sequence identity is not an important contributor to the score. Similar figures for the score dependency on GC-content can be seen in Additional file 1: Figures S12 (training dataset) and S13 (test dataset). These figures show that the score is not highly dependent on the GC-content. Hence, it is not necessary to take sequence identity or GC-content into account when the score is evaluated. The fluctuations especially at the high and low ranges are likely to be due to a low number of data points.

Next, the discriminative performance of DotcodeR on the test set was investigated by comparing DotcodeR and RNApdist [24]. RNApdist is designed to calculate distances between RNA secondary structure ensembles, which can be obtained by aligning profile vectors created from dot plots. Figure 3 illustrates ROC curves for these two approaches. As it can be seen, DotcodeR outperforms RNApdist, which is not surprising since RNApdist was designed with other purposes in mind. ROC curves for respective methods and families can be found in Additional file 1: Figures S6–S9.

DotcodeR’s and RNApdist’s run-time and memory usage were measured on a family named IRES_Picorna, which needed the longest computation time among all families in the test set. Table 2 indicates that DotcodeR runs significantly faster for this type of comparison, whereas both methods have similar memory consumption.

**Table 2:** Run-time of each comparative method on the IRES_Picorna family of length 253 nt in the test set of simulated short genomes

Method	# of processors used	Time (s)	Memory (MB)
DotcodeR	1	**6.00**	**4.46**
RNApdist	1	144.55	4.60

Note that these results are only from real-against-real window comparison. The best performance is shown in bold face. This test was serially done on a server with Intel Xeon CPU 2.00 GHz with 8 cores and 65 GB memory

Additional file 1: Figure S10 shows the effect of window size (size: 50 or 120 nt) and step size (size: 10 or 30 nt). A window size of 120 nt is better than a smaller window size of 50 nt. The window size of 120 nt fits very well with the length distribution of the structures in Rfam and has also been found to be optimal for other methods based on fixed window sizes (RNAz [18] for example). A smaller window size of 50 nt was tested to see if it would give a better signal as it makes it more likely to find a window that only overlaps an RNA structure rather than a mixture of RNA structures and unstructured sequences. A larger window size would add more unstructured sequences to most windows due to the Rfam length distribution, and longer RNAs are likely to be found as series of windows predicted to be RNA. ncRNAs longer than 120 nt would be expected to show up as stretches of high scoring windows. Additional file 1: Figure S16 shows the performance for step sizes of 10, 30 and 50 nt. Here, a smaller step size unsurprisingly leads to better performance, but this better performance comes at the cost of increasing the run-time. Step size of 30 nt is nine times faster than step size of 10 nt, but 2.8 times slower than step size of 50 nt. The figure shows that a step size of 30 nt provides a good trade-off between speed and performance.

Additional file 1: Figure S14 shows the DotcodeR score as a function of the offset between the two structures for the real-against-real sequences in the test set. The figure shows that the DotcodeR score is highest when the two structures are not offset or offset by one, which is *d* used in the screen. Additional file 1: Figure S15 shows ROC curves for the training and test datasets using different values of *d*.

### Genomic screen for structured RNAs

#### Computational performance

A genomic screen was performed on the two cleaned chromosomes described in the ‘Genomic sequences’ section. Table 3 shows that DotcodeR reduced the search space to 2.9% compared to a naïve scan that takes all possible pairs of windows into account in the cleaned input. Run-time for the chromosomal screen by DotcodeR on the cleaned input is 10.2 CPU months or approximately less than three days on a small computer cluster. When considering a screen of the full genomes of size 3G bases, the run-time of DotcodeR is estimated to be 925 CPU years or 8.6 years on the same small computer cluster (see also Additional file 1: Subsections S3.2 and S3.3 for details). Although this number may be huge, with the use of a large computer cluster the application of the method to two full genomes would be feasible.

**Table 3:** The comparison between the number of all possible pairs of windows in input and that of pairs of windows in DotcodeR prediction on human chromosome 21 and mouse chromosome 19

# of pairs of windows in original input	# of pairs of windows in cleaned input	# of pairs of windows in prediction	Reduction (%)
3.18×10^12^	2.30×10^12^	6.75×10^10^	97.07

The cleaned input was generated by removing known repeat and aligned regions from the original input. As for the prediction, we counted only the windows with score of at least 20. Reduction is one minus fraction of the number of pairs of windows in prediction to that of pairs of windows in cleaned input

#### Predictive performance

To evaluate predictive performance, the predictions of DotcodeR were compared to the annotations of the chromosomes provided by Ensembl release 85. Each exon in the reference annotation corresponds to exactly one window, namely the one with the biggest overlap to the respective exon. The sensitivity is calculated, where TP is the number of exon pairs that share the same ncRNA biotype, and FN is the number of exon pairs in the reference that were not predicted. It should be noted that these pairs between human and mouse are the same ncRNA biotype (e.g. miRNA–miRNA) because it is assumed that two regions to be compared are structurally similar and thus they are expected to belong to the same family if they have the same biotype. Additional file 1: Table S3 shows gene and transcript biotypes used to annotate the predicted regions. The annotation of the ncRNAs in Ensembl ranges from very specific RNAs like miRNA, snoRNA, snRNA and rRNA, each of which contains a few structured RNAs, over misc RNA, which is usually structured RNAs but from very mixed families, to broad categories like lincRNA, processed transcript (transcript biotype: lincRNA or processed transcript) and sense intronic, which may or may not be structured at all. It is to be noted that snoRNAs can be further classified into H/ACA box and C/D box, each of which is known to form a different structure.

It is necessary to consider the strand on which the RNA structures are located, since G-U is allowed to form a base pair, but its complement C-A is not. There are three cases of strand combinations in annotation, i.e. positive-against-positive, positive-against-negative and negative-against-negative. When evaluating performance, the fourth case of negative-against-positive is expected to be the same as the positive-against-negative case (RNAs on opposite strands), and therefore these two cases are grouped as one.

**Fig. 5:**
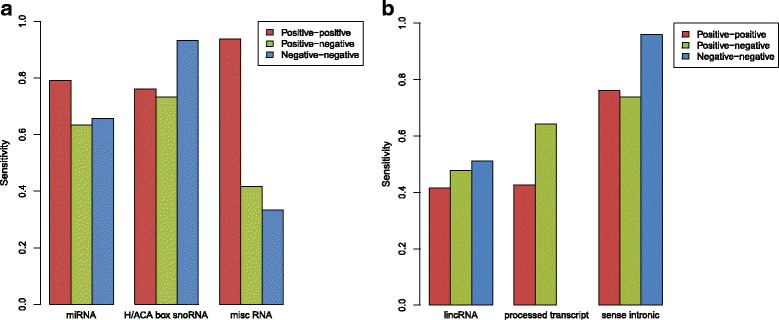
Sensitivity in predicting ncRNAs on the pair of human chromosome 21 and mouse chromosome 19 using DotcodeR. The left panel shows the performance for the ncRNAs that are known to be structured (**a**), whereas the right panel is for the ncRNAs that might be or might not be structured (**b**). DotcodeR was applied only to the positive strand of each chromosome, and the performance on the negative strand actually comes from comparison between predicted regions on the corresponding positive strand and annotated regions on the negative strand. Note that sensitivity for rRNA was removed from the figure due to its low number of annotated genes pairs, and there are no cases of processed transcripts on the negative–negative strand. The sensitivity for C/D box snoRNA was also removed because two of the three cases have no annotated pairs. A single missed case of snRNA is not shown. Details of sensitivity are shown in Additional file 1: Table S4

Figure 5a and the corresponding numbers in Additional file 1: Table S4 indicate that the sensitivity of 0.83 is obtainable for the three known structured ncRNA families (miRNA of sensitivity 0.79, H/ACA box snoRNA of sensitivity 0.76 and misc RNA of sensitivity 0.94) on the positive–positive strand. Whereas this is below 0.9 selected in the training set, it is still high enough for the method to be used as a pre-filter. The results of the RNAs in Fig. 5 also indicate that a scan that includes the positive-against-negative strand and the negative-against-negative one would significantly improve the predictions. However, this would come at the cost of a four times longer run-time.

The sensitivity for C/D box snoRNAs is significantly worse than that for H/ACA box snoRNAs (see Additional file 1: Table S4). This could be due to the fact that the correct secondary structure of C/D box snoRNAs with a very long loop is hard to predict [40, 41]. This effect is also visible in Additional file 1: Figures S1 and S2, where C/D box snoRNA families (SNORD) tend to perform worse than H/ACA box snoRNA families (SNORA).

For lincRNAs and processed transcripts (gene biotype: lincRNA and processed transcript), the sensitivity is low compared to the known structured RNAs. The function and the structure of most lincRNAs are currently not known, and the lack of detectable structure is therefore not surprising. The sense intronic transcripts are transcripts located inside the introns of other genes. It is well known that many introns harbor structured RNAs.

## Discussion

The sensitivity for the genomic screen on the human and mouse chromosomes is poorer than that on the artificial short genomes. This is not surprising since the evaluation for the artificial short genomes is limited to comparing sequences with the same RNA structure as these were build from Rfam clans. In the real annotation, the structures are much more diverse as the annotated RNA structures are much broader than in Rfam. Furthermore, using shuffled sequences in the benchmarking, though necessary, may lead to an overestimation of the performance on the benchmark data. Considering these observations, we consider a sensitivity of 83% in the genomic screen good enough for a pre-screen.

DotcodeR seeks to detect structured RNAs conserved between two genomic sequences, but it should be noted that there are a few limitations in this method. First, DotcodeR has the parameter of window size *w* for genomic screen, which restricts the length of the RNA secondary structure that DotcodeR can predict. DotcodeR is also expected to have problems detecting RNA structures that span more than one exon. Taking transcripts rather than genomic sequences as input could solve this problem, but the method has not been tested on this kind of data. Thirdly, the presented method is meant to be a pre-filter for genomic screen and thus having a high false positive rate is acceptable.

The remaining pipeline after running DotcodeR could be:

1. Rerun DotcodeR with a step size of 10 and a very high sensitivity using the output of the current scan as input;
2. Then use a structural alignment method optimized for finding local structural alignments like Foldalign [8];
3. Finally cluster the results with GraphClust [29] or RNAscClust [30] to find families of structures. A method like Structator [31] could be used to search for missed members of the clusters.

The high false positive rate slows down further analysis, but a second round of DotcodeR with smaller step size could reduce the number of false positive windows, while keeping sensitivity high. The false negative rate is a bigger problem since DotcodeR is intended as a pre-filter, and any RNAs missed in this step will not be recovered by later steps in a pipeline. However, since most RNAs are likely to be parts of larger families, a single missed pair of RNAs may not be a problem because the RNAs involved may be found in pairs with other members of the same family.

## Conclusions

Genomic screens for structured RNAs play an essential role in the annotation of ncRNAs that have not yet been annotated in public data. To this end, the algorithm DotcodeR for *de novo* prediction of structured RNAs in genomic sequences was presented. It is based on comparative structural information and uses a coarse-grained secondary structure dot plots to predict if structures are similar or not. *In silico* experimental results showed that DotcodeR was able to discard genomic regions irrelevant to structured RNAs while keeping a good number of existing ncRNA regions on the pair of real chromosome sequences. This means that DotcodeR would be useful as a pre-filter in the process of *de novo* prediction of ncRNAs.

As the first step in the *de novo* prediction, DotcodeR can be used to quickly and efficiently select ncRNA candidates from a large number of subsequence pairs between two genomes. The method has high false positive rate, but still reduces the number of window combinations with 97.1% compared to the input set. A next step in the *de novo* prediction pipeline could be to cluster the DotcodeR predictions, so that the slower rigorous methods used in the third step would only have to be used within each cluster. Clustering step could also help determining the function of the predicted structure.

## Additional file

Additional file 1 Supplementary Material is available online. (PDF 293 kb)

## References

[1] Weirick T, Militello G, Müller R, John D, Dimmeler S, Uchida S (2016). The identification and characterization of novel transcripts from RNA-seq data. Brief Bioinform.

[2] Gorodkin J, Hofacker IL, Torarinsson E, Yao Z, Havgaard JH, Ruzzo W (2010). De novo prediction of structured RNAs from genomic sequences. Trends Biotechnol.

[3] Gardner P, Giegerich R (2004). A comprehensive comparison of comparative RNA structure prediction approaches. BMC Bioinformatics.

[4] Sankoff J (1985). Simultaneous solution of the RNA folding, alignment and protosequence problems. SIAM J Appl Math.

[5] Gorodkin J, Heyer LJ, Stormo GD (1997). Finding the most significant common sequence and structure motifs in a set of RNA sequences. Nucleic Acids Res.

[6] Torarinsson E, Sawera M, Havgaard JH, Fredholm M, Gorodkin J (2006). Thousands of corresponding human and mouse genomic regions unalignable in primary sequence contain common RNA structure. Genome Res.

[7] Havgaard JH, Torarinsson E, Gorodkin J (2007). Fast pairwise structural RNA alignments by pruning of the dynamical programming matrix. PLoS Comput Biol.

[8] Sundfeld D, Havgaard JH, de Melo MA, Gorodkin J (2016). Foldalign 2.5: multithreaded implementation for pairwise structural RNA alignment. Bioinformatics.

[9] Mathews DH, Turner DH (2002). Dynalign: an algorithm for finding the secondary structure common to two RNA sequences. J Mol Biol.

[10] Uzilov AV, Keegan JM, Mathews DH (2006). Detection of non-coding RNAs on the basis of predicted secondary structure formation free energy change. BMC Bioinformatics.

[11] Fu Y, Xu ZZ, Lu ZJ, Zhao S, Mathews DH (2015). Discovery of novel ncRNA sequences in multiple genome alignments on the basis of conserved and stable secondary structures. PLoS ONE.

[12] Yao Z, Weinberg Z, Ruzzo W (2006). CMfinder–a covariance model based RNA motif finding algorithm. Bioinformatics.

[13] Will S, Reiche K, Hofacker IL, Stadler PF, Backofen R (2007). Inferring noncoding RNA families and classes by means of genome-scale structure-based clustering. PLoS Comput Biol.

[14] Will S, Joshi T, Hofacker IL, Stadler PF, Backofen R (2012). LocARNA-P: accurate boundary prediction and improved detection of structural RNAs. RNA.

[15] Kiryu H, Tabei Y, Kin T, Asai K (2007). Murlet: a practical multiple alignment tool for structural RNA sequences. Bioinformatics.

[16] Do CB, Foo CS, Batzoglou S (2008). A max-margin model for efficient simultaneous alignment and folding of RNA sequences. Bioinformatics.

[17] Sato K, Kato Y, Akutsu T, Asai K, Sakakibara Y (2012). DAFS: simultaneous aligning and folding of RNA sequences via dual decomposition. Bioinformatics.

[18] Washietl S, Hofacker IL, Stadler PF (2005). Fast and reliable prediction of noncoding RNAs. Proc Natl Acad Sci USA.

[19] Pedersen JS, Bejerano G, Siepel A, Rosenbloom K, Lindblad-Toh K, Lander ES (2006). Identification and classification of conserved RNA secondary structures in the human genome. PLoS Comput Biol.

[20] Seemann S, Gorodkin J, Backofen R (2008). Unifying evolutionary and thermodynamic information for RNA folding of multiple alignments. Nucleic Acids Res.

[21] Torarinsson E, Yao Z, Wiklund ED, Bramsen JB, Hansen C, Kjems J (2008). Comparative genomics beyond sequence-based alignments: RNA structures in the ENCODE regions. Genome Res.

[22] Gardner PP, Wilm A, Washietl S (2005). A benchmark of multiple sequence alignment programs upon structural RNAs. Nucleic Acids Res.

[23] Hofacker IL, Fontana W, Stadler PF, Bonhoeffer S, Tacker M, Schuster P (1994). Fast folding and comparison of RNA secondary structures. Monatsh Chem.

[24] Lorenz R, Bernhart SH, zu Siederdissen CH, Tafer H, Flamm C, Stadler PF (2011). ViennaRNA package 2.0. Algorithms Mol Biol.

[25] Agius P, Bennett KP, Zuker M (2010). Comparing RNA secondary structures using a relaxed base-pair score. RNA.

[26] Ivry T, Michal S, Avihoo A, Sapiro G, Barash D (2009). An image processing approach to computing distances between RNA secondary structure dot plots. Algorithms Mol Biol.

[27] Tsang HH, Jacob C. RNADPCompare: an algorithm for comparing RNA secondary structures based on image processing techniques. In: Proceedings of the IEEE Congress on Evolutionary Computation (CEC): 5-8 June 2011. New Orleans: 2011. p. 1288–95.

[28] Churkin A, Barash D (2013). RNA dot plots: an image representation for RNA secondary structure analysis and manipulations. WIREs RNA.

[29] Heyne S, Costa F, Rose D, Backofen R (2012). GraphClust: alignment-free structural clustering of local RNA secondary structures. Bioinformatics.

[30] Miladi M, Junge A, Costa F, Seemann SE, Havgaard JH, Gorodkin J, Backofen R (2017). RNAscClust: clustering RNA sequences using structure conservation and graph based motifs. Bioinformatics.

[31] Meyer F, Kurtz S, Backofen R, Will S, Beckstette M (2011). Structator: fast index-based search for RNA sequence-structure patterns. BMC Bioinformatics.

[32] McCaskill JS (1990). The equilibrium partition function and base pair binding probabilities for RNA secondary structure. Biopolymers.

[33] Bernhart SH, Hofacker IL, Stadler PF (2006). Local RNA base pairing probabilities in large sequences. Bioinformatics.

[34] Nawrocki EP, Burge SW, Bateman A, Daub J, Eberhardt RY, Eddy SR (2015). Rfam 12.0: updates to the RNA families database. Nucleic Acids Res.

[35] Cunningham F, Amode MR, Barrell D, Beal K, Billis K, Brent S (2015). Ensembl 2015. Nucleic Acids Res.

[36] Jiang M, Anderson J, Gillespie J, Mayne M (2008). uShuffle: a useful tool for shuffling biological sequences while preserving the k-let counts. BMC Bioinformatics.

[37] Speir ML, Zweig AS, Rosenbloom KR, Raney BJ, Paten B, Nejad P (2016). The UCSC Genome Browser database: 2016 update. Nucleic Acids Res.

[38] Schwartz S, Kent WJ, Smit A, Zhang Z, Baertsch R, Hardison RC (2003). Human–mouse alignments with BLASTZ. Genome Res.

[39] Anandam P, Torarinsson E, Ruzzo WL (2009). Multiperm: shuffling multiple sequence alignments while approximately preserving dinucleotide frequencies. Bioinformatics.

[40] Tafer H, Kehr S, Hertel J, Hofacker IL, Stadler PF (2010). RNAsnoop: efficient target prediction for H/ACA snoRNAs. Bioinformatics.

[41] Bartschat S, Kehr S, Tafer H, Stadler PF, Hertel J (2014). snoStrip: a snoRNA annotation pipeline. Bioinformatics.

