# Supplementary material for: Alignment-free comparative genomic screen for structured RNAs using coarse-grained secondary structure dot plots
Source: BMC Genomics. 2017 Dec 2;18:935. doi: 10.1186/s12864-017-4309-y (PMC5712110; doi:10.1186/s12864-017-4309-y)
Supplement: Supplementary file 1 — Supplementary Material is available online. (PDF 293 kb) [file 12864_2017_4309_MOESM1_ESM.pdf]

Supplementary Material on "Alignment-free comparative genomic screen for structured RNAs using coarse-grained secondary structure dot plots"

Yuki Kato[1,*], Jan Gorodkin[2] & Jakob Hull Havgaard[2,*]

1 Department of RNA Biology and Neuroscience, Graduate School of Medicine, Osaka University, Japan
2 Center for non-coding RNA in Technology and Health (RTH), University of Copenhagen, Denmark

[*]Correspondence should be addressed to Y. K. (ykato@rna.med.osaka-u.ac.jp) or J. H. H. (hull@rth.dk)

Contents

S1 Supplementary Tables **3**

S2 Supplementary Figures **7**

S3 Supplementary Notes **23**
- S3.1 Parameter settings . . . 23
- S3.2 Calculating the number of pairs of windows in input . . . 23
- S3.3 Estimating run-time for chromosomal screen and genomic screen . . . 23
- S3.4 Criterion of determining repeat and aligned regions in annotation . . . 24

List of Tables

- S1 Rfam families included in the training set . . . 3
- S2 Rfam families included in the test set . . . 4
- S3 Details of ncRNA biotypes used to annotate predicted regions in genomic screen . . . 5
- S4 Sensitivity for ncRNA detection on the pair of human chromosome 21 and mouse chromosome 19 using DotcodeR . . . 6

List of Figures

- S1 ROC curves that show discriminative power of DotcodeR on snoRNAs in the training set of simulated short genomes (gene-shuffled) . . . 7
- S2 ROC curves that show discriminative power of DotcodeR on snoRNAs in the training set of simulated short genomes (genome-shuffled) . . . 8
- S3 ROC curves for DotcodeR on respective RNA families in the training set of simulated short genomes (gene-shuffled) . . . 9
- S4 ROC curves for DotcodeR on respective RNA families in the training set of simulated short genomes (genome-shuffled) . . . 10
- S5 ROC curves that show discriminative power of DotcodeR on the training set of simulated short genomes . . . 11
- S6 ROC curves for DotcodeR on respective RNA families in the test set of simulated short genomes (gene-shuffled) . . . 12
- S7 ROC curves for DotcodeR on respective RNA families in the test set of simulated short genomes (genome-shuffled) . . . 13
- S8 ROC curves for RNApdist on respective RNA families in the test set of simulated short genomes (gene-shuffled) . . . 14
- S9 ROC curves for RNApdist on respective RNA families in the test set of simulated short genomes (genome-shuffled) . . . 15
- S10 ROC curves that show discriminative power of DotcodeR and RNApdist on the test set of simulated short genomes . . . 16
- S11 DotcodeR score as a function of sequence identity on the training set of simulated short genomes 17
- S12 DotcodeR score as a function of GC content on the training set of simulated short genomes . . 18
- S13 DotcodeR score as a function of GC content on the test set of simulated short genomes 19
- S14 DotcodeR score as a function of window offset between two similar structures . . . 20
- S15 ROC curves for DotcodeR with $d \in \{1, 5, 10, 15, 20\}$ on the benchmark data . . . 21
- S16 ROC curves for DotcodeR with the step size $s \in \{10, 30, 50\}$ on the benchmark data 22

S1 Supplementary Tables

Table S1: Rfam families included in the training set.

Family ID	# of sequences	Family ID	# of sequences
U1	5	MIR480	5
U2	5	MIR807	5
Vault	5	mir-1302	5
U4	5	PK-G12rRNA	5
U5	5	PhotoRC-II	5
U6	5	SAM-II_long_loops	5
let-7	5	SAM-SAH	5
SECIS_1	5	msiK	5
mir-2	5	pan	5
mir-7	5	wcaG	5
SNORA71	5	ykkC-III	5
TPP	5	THF	5
U7	5	SmY	5
mir-1	5	mir-1937	5
SNORD116	5	MIR2907	5
S15	5	sRNA-Xcc1	5
t44	5	tRNA	4
mir-192	5	RNaseP_bact_a	5
mir-199	5	Bacteria_small_SRP	5
SNORA70	5	SAM	4
Purine	5	SAM-I-IV-variant	1
mir-9	5	SNORA13	5
mir-124	5	SNORA17	5
mir-TAR	5	SNORA26	5
ydaO-yuaA	5	SNORD36	3
ykoK	5	SNORD33	5
ykkC-yxkD	5	SNORD113	5
serC	5	mir-449	5
SAM_alpha	5	mir-36	5
PreQ1	5	mir-216	5
Prion_pknot	5	mir-290	1
mir-154	5	mir-515	2
mir-204	5	mir-302	2
mir-184	5	MIR171_1	5
mir-33	5	U3	5
mir-147	5	suhB	5
mir-320	5		

Table S2: Rfam families included in the test set.

Family ID	# of sequences	Family ID	# of sequences
5S_rRNA	5	Downstream-peptide	5
5_8S_rRNA	5	Flavo-1	5
6S	5	JUMPstart	5
Histone3	5	crcB	5
IRE_I	5	glnA	5
FMN	5	pfl	5
IRES_Picorna	5	ASdes	5
HIV_GSL3	5	ASpks	5
Entero_5_CRE	5	c-di-GMP-II	5
HCV_SLVII	5	ffh	5
HCV_SLIV	5	GABA3	5
HIV_FE	5	IMES-2	5
K_chan_RES	5	ar14	5
L10_leader	5	sau-50	5
c-di-GMP-I	5	PYLIS_3	5
mini-ykkC	5	AdoCbl-variant	1
isrK	5	Cobalamin	4
HIV_POL-1_SL	5	group-II-D1D4-3	1
IsrR	5	group-II-D1D4-7	1
mascRNA-menRNA	5	group-II-D1D4-1	1
Acido-Lenti-1	5	group-II-D1D4-6	1
Bacillaceae-1	5	group-II-D1D4-2	1
C4	5	TwoAYGGAY	5
Cyano-1	5		

Table S3: Details of ncRNA biotypes used to annotate predicted regions in genomic screen, which were taken from the GTF annotation files available in the Ensembl database.

Gene biotype	Transcript biotype
3prime overlapping ncrna	3prime overlapping ncrna
lincRNA	lincRNA
miRNA	miRNA
misc RNA	misc RNA
processed transcript	lincRNA
processed transcript	processed transcript
rRNA	rRNA
sense intronic	sense intronic
sense overlapping	sense overlapping
snRNA	snRNA
snoRNA	snoRNA

Table S4: Sensitivity for non-coding RNA (ncRNA) detection on the pair of human chromosome 21 and mouse chromosome 19 using DotcodeR. TP and FN denote the number of true positives and that of false negatives, respectively, whose definitions are described in Section 3.3 in the main article. Note that ncRNA type with sensitivity of N/A is not shown in the bar plot in Figure 5 in the main article. The result of snRNA is not also shown in Figure 5 as it consists of just one example.

	positive–positive			positive–negative			negative–negative		
ncRNA type	TP	TP+FN	Sensitivity	TP	TP+FN	Sensitivity	TP	TP+FN	Sensitivity
miRNA	247	312	0.792	173	273	0.634	69	105	0.657
rRNA	0	0	N/A	3	4	0.750	0	0	N/A
H/ACA box snoRNA	32	42	0.762	77	105	0.733	56	60	0.933
C/D box snoRNA	0	16	0	0	0	N/A	0	0	N/A
snRNA	0	0	N/A	0	0	N/A	0	1	0
misc RNA	15	16	0.938	15	36	0.417	3	9	0.333
lincRNA	16638	40040	0.416	20357	42588	0.478	23432	45747	0.512
processed transcript	644	1512	0.426	712	1107	0.643	0	0	N/A
sense intronic	35	46	0.761	153	207	0.739	164	171	0.959

S2 Supplementary Figures

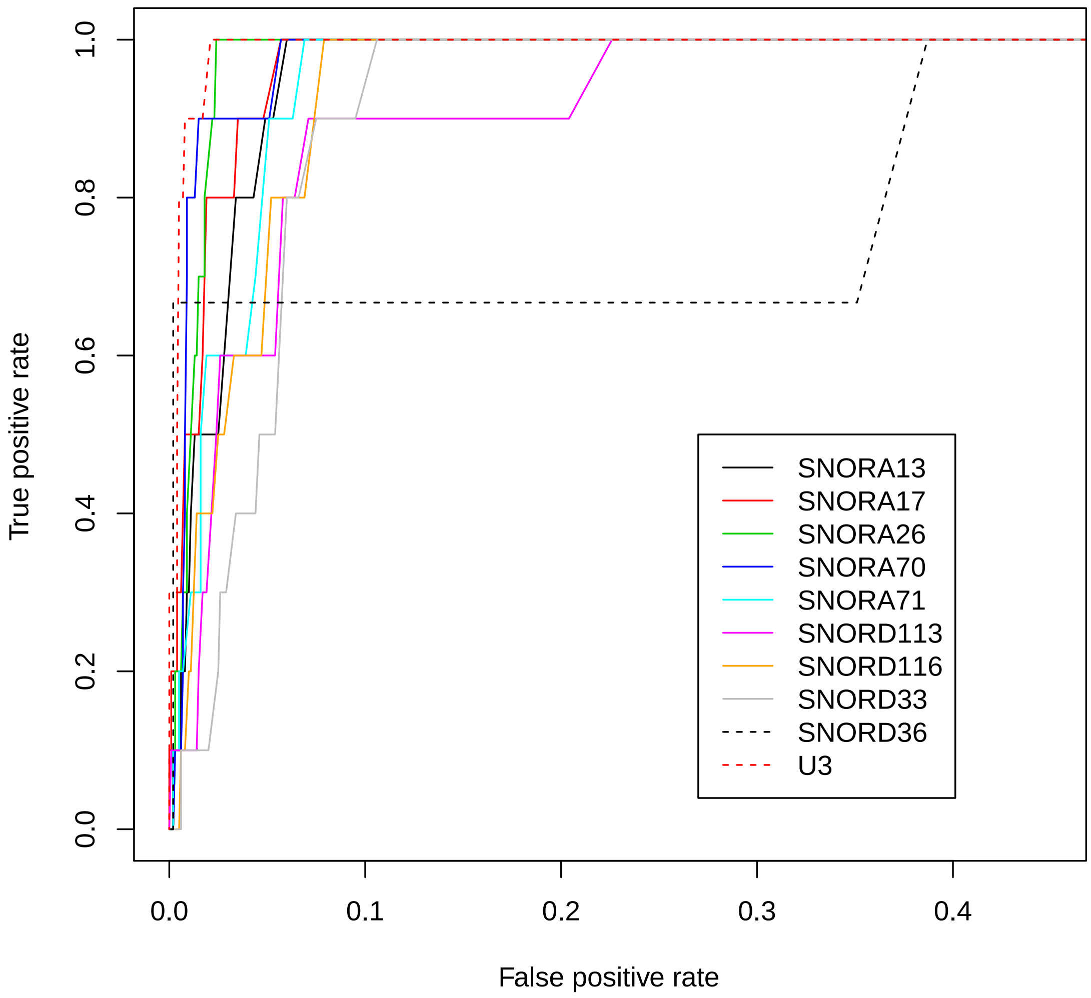

Figure S1: Receiver operating characteristic (ROC) curves that show discriminative power of DotcodeR on snoRNAs in the training set of simulated short genomes whose negatives are 'gene-shuffled' sequences. In this test, we used the window size of 120 nt, the step size of 30 nt and $d = 1$.

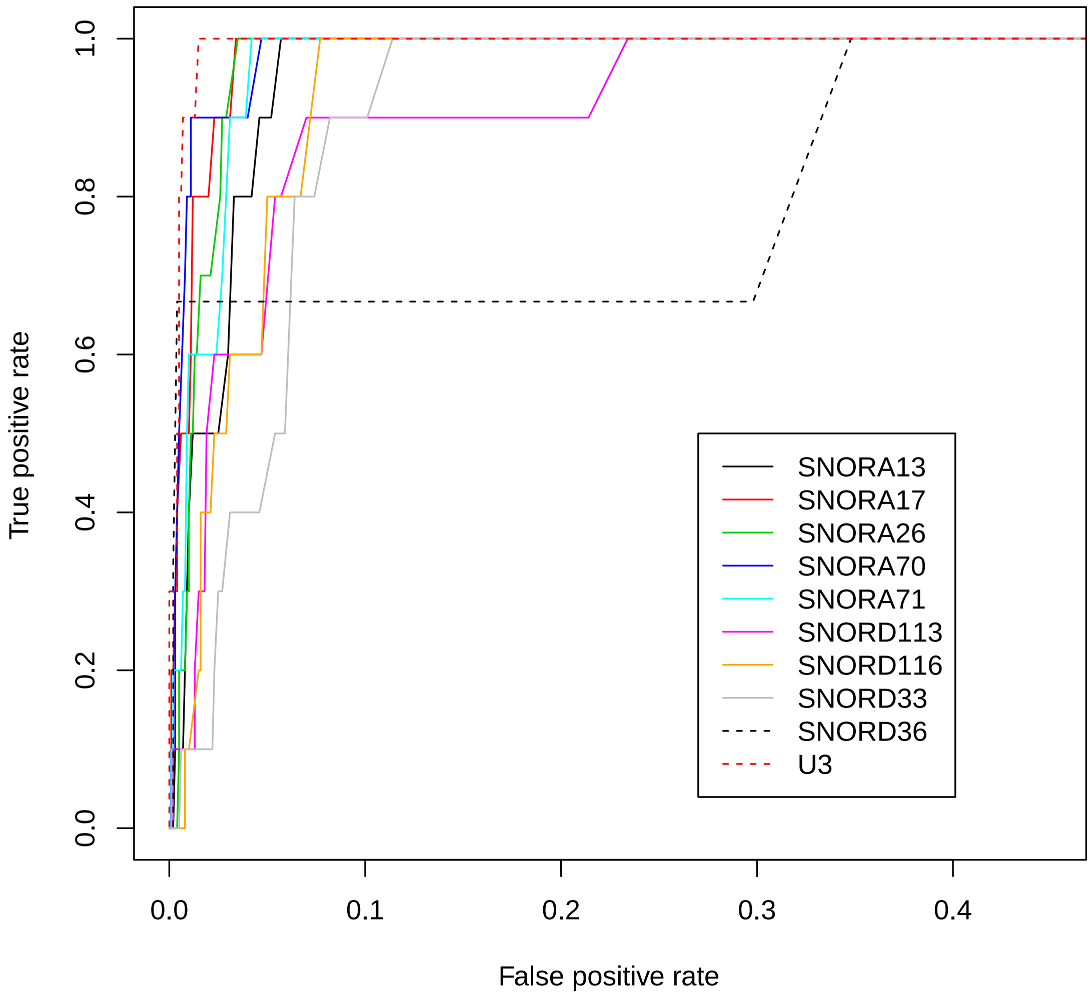

Figure S2: ROC curves that show discriminative power of DotcodeR on snoRNAs in the training set of simulated short genomes whose negatives are 'genome-shuffled' sequences. In this test, we used the window size of 120 nt, the step size of 30 nt and $d = 1$.

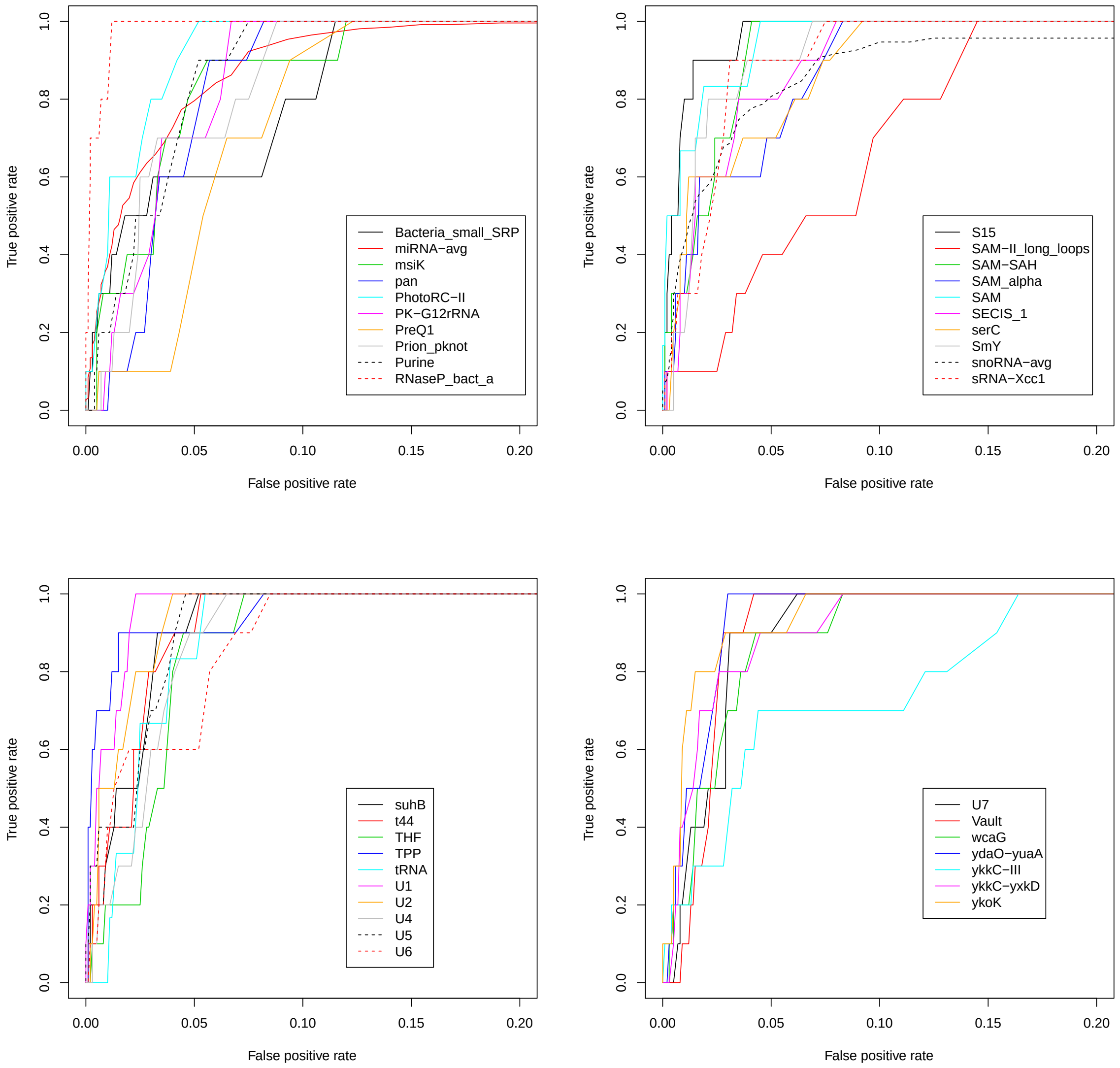

Figure S3: ROC curves for DotcodeR on respective RNA families in the training set of simulated short genomes whose negatives are ‘gene-shuffled’ sequences. In this test, we used the window size of 120 nt, the step size of 30 nt and $d = 1$.

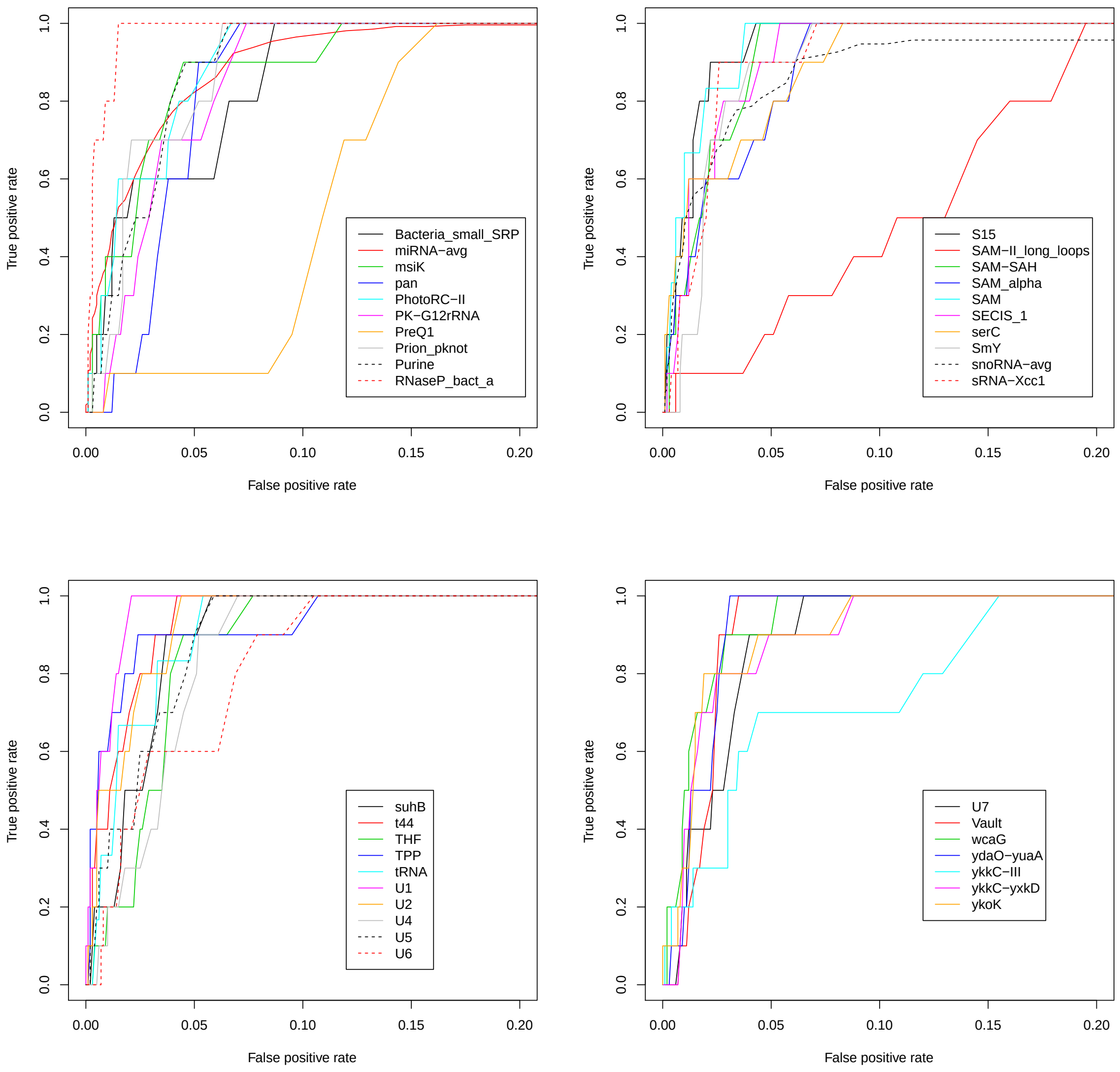

Figure S4: ROC curves for DotcodeR on respective RNA families in the training set of simulated short genomes whose negatives are ‘genome-shuffled’ sequences. In this test, we used the window size of 120 nt, the step size of 30 nt and $d = 1$.

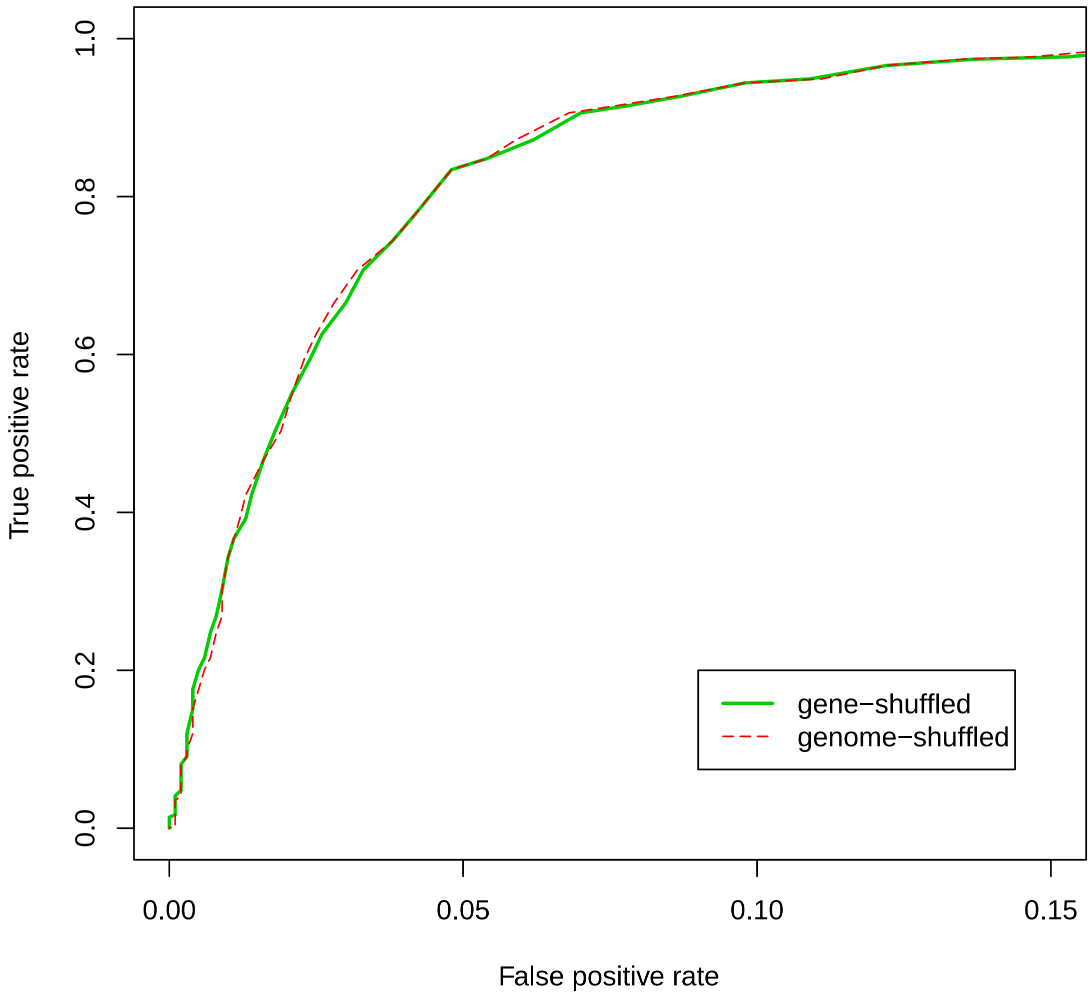

Figure S5: ROC curves that show discriminative power of DotcodeR on the training set of simulated short genomes. In this test, we used the window size of 120 nt, the step size of 30 nt and $d = 1$. Note that accuracy was calculated by averaging over all results of the families in the dataset.

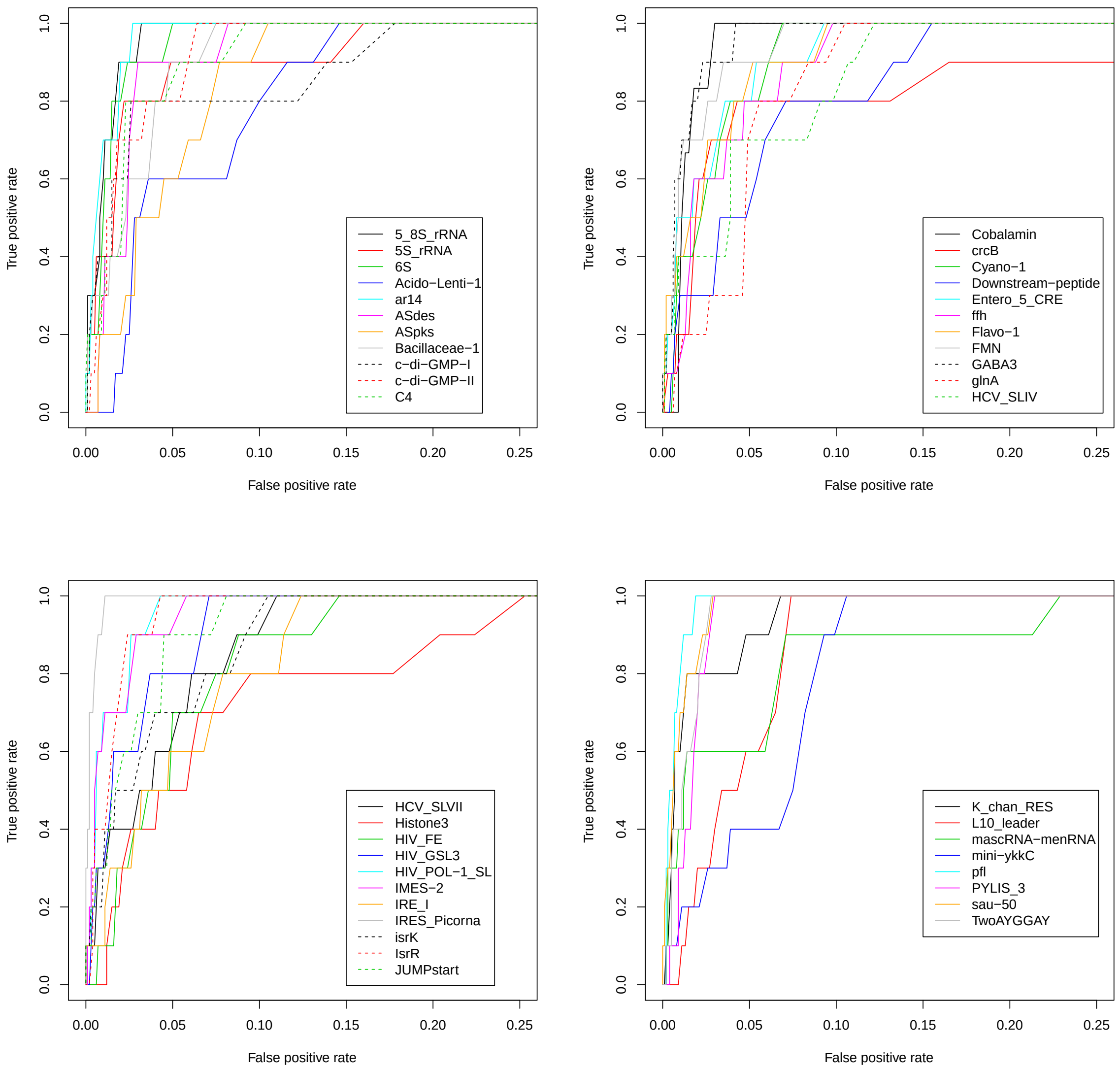

Figure S6: ROC curves for DotcodeR on respective RNA families in the test set of simulated short genomes whose negatives are 'gene-shuffled' sequences. In this test, we used the window size of 120 nt, the step size of 30 nt and $d = 1$.

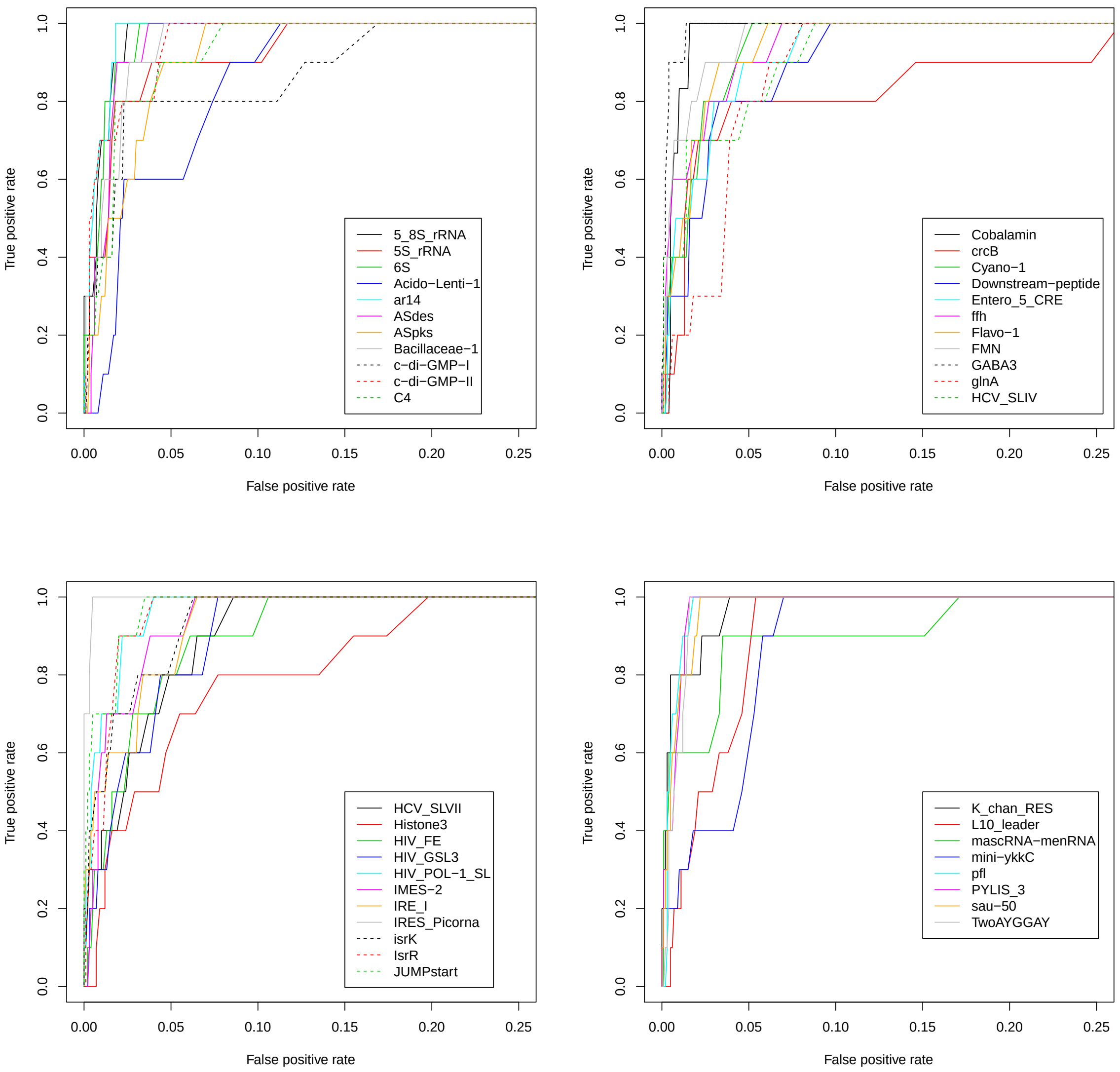

Figure S7: ROC curves for DotcodeR on respective RNA families in the test set of simulated short genomes whose negatives are ‘genome-shuffled’ sequences. In this test, we used the window size of 120 nt, the step size of 30 nt and $d = 1$.

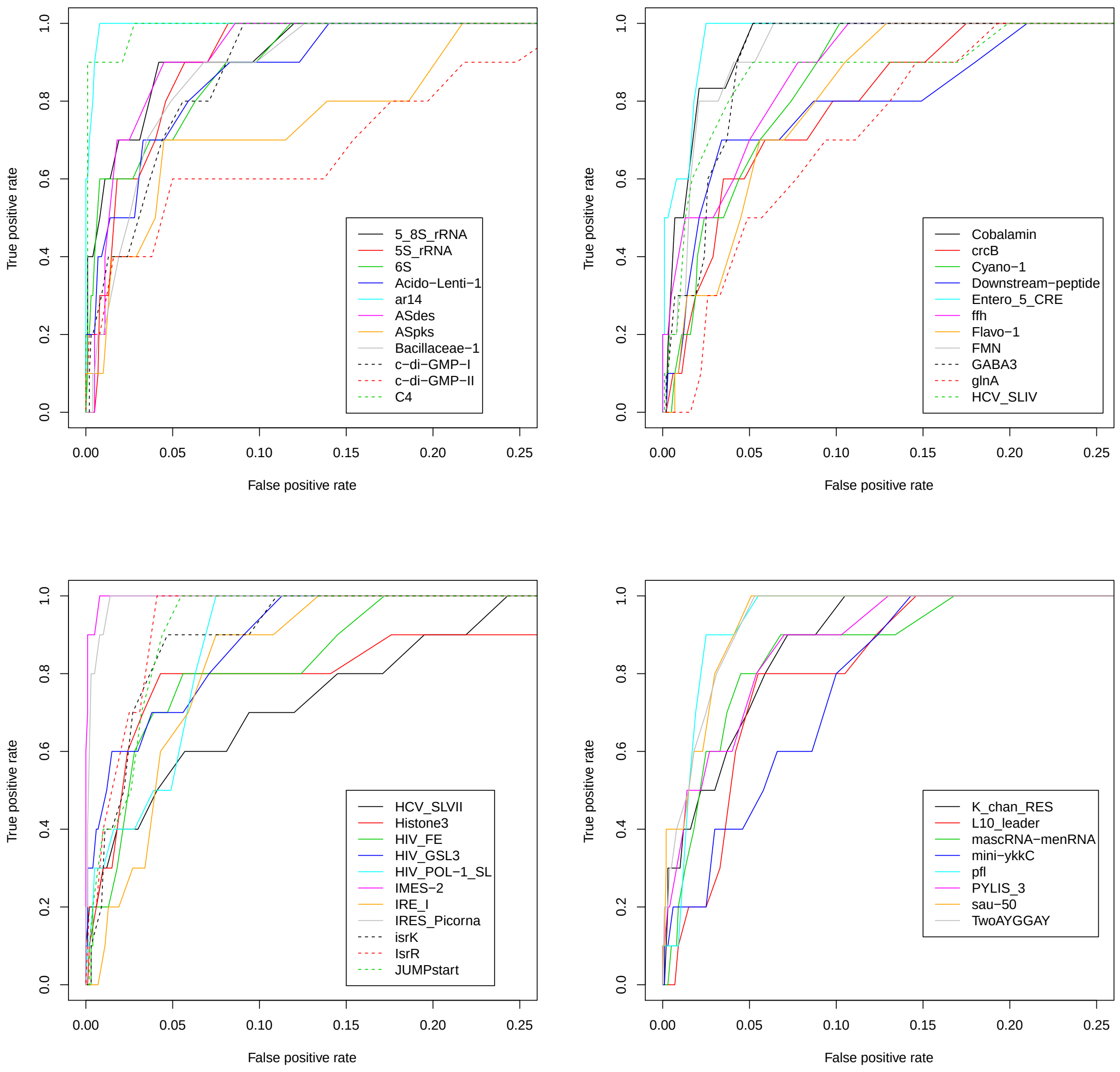

Figure S8: ROC curves for RNApdist on respective RNA families in the test set of simulated short genomes whose negatives are ‘gene-shuffled’ sequences. In this test, we used the window size of 120 nt and the step size of 30 nt.

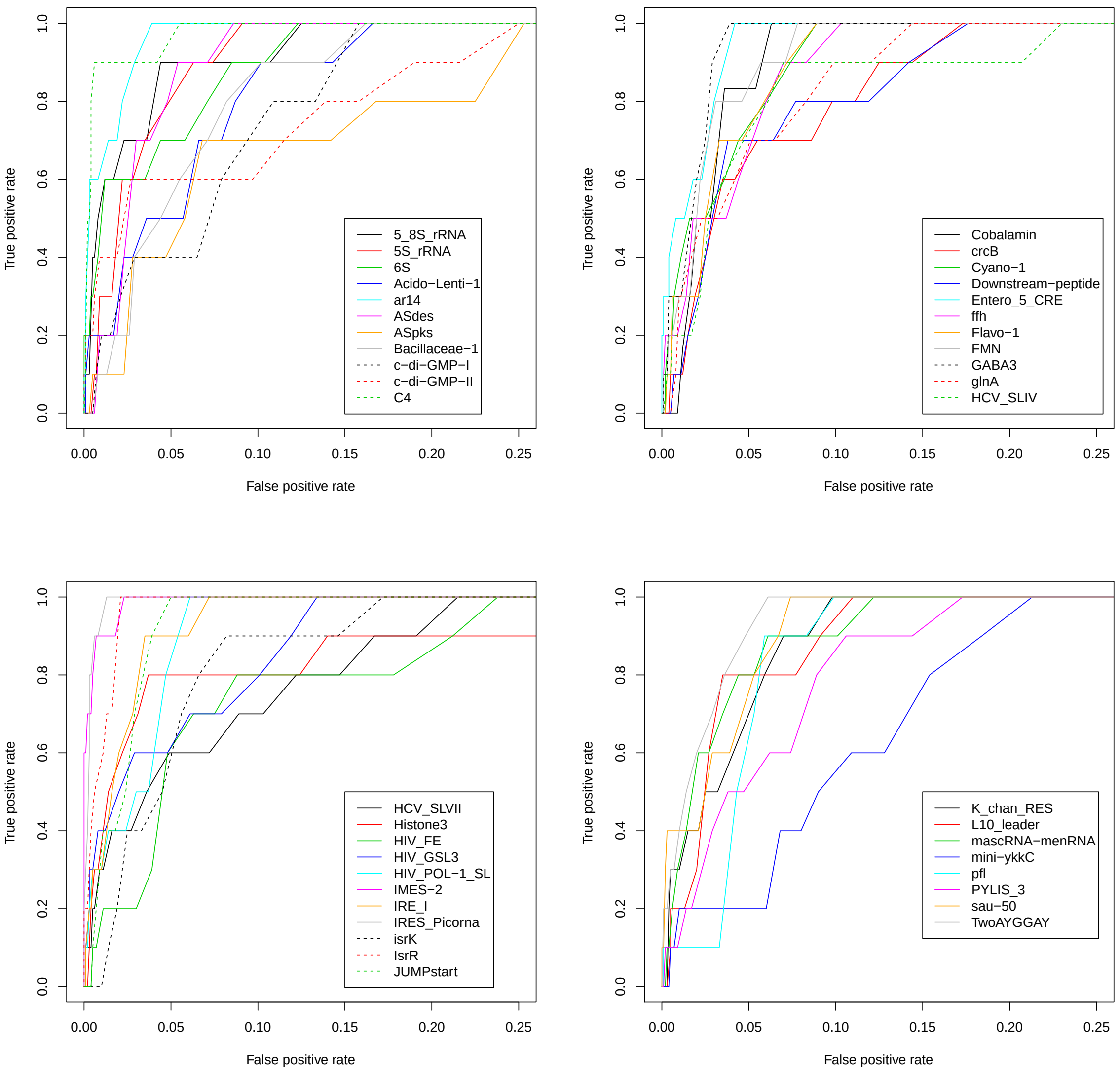

Figure S9: ROC curves for RNApdist on respective RNA families in the test set of simulated short genomes whose negatives are ‘genome-shuffled’ sequences. In this test we used the window size of 120 nt and the step size of 30 nt.

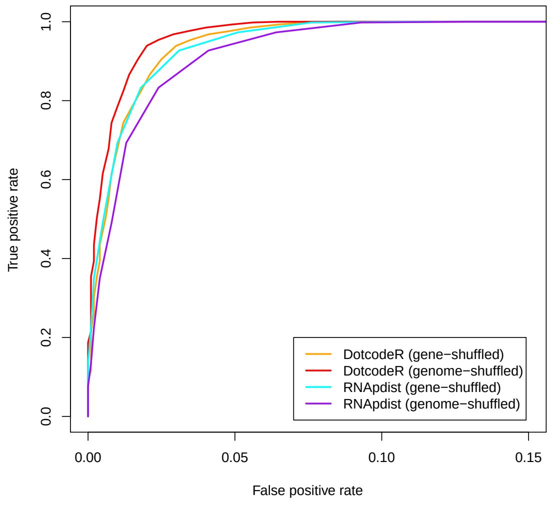

(a) Window size 50, step size 10

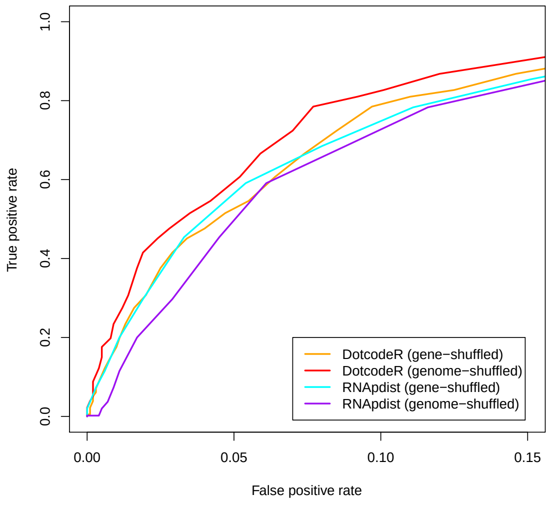

(b) Window size 50, step size 30

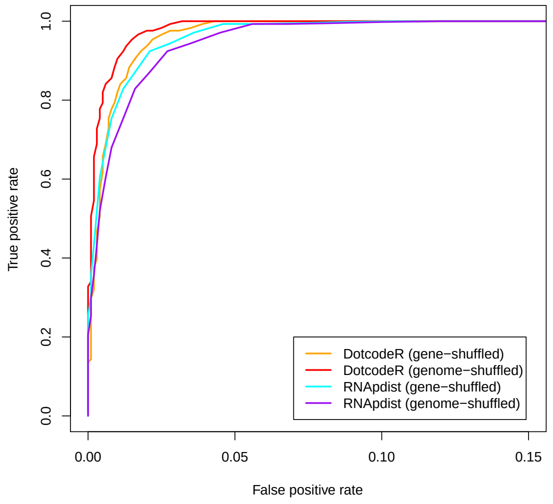

(c) Window size 120, step size 10

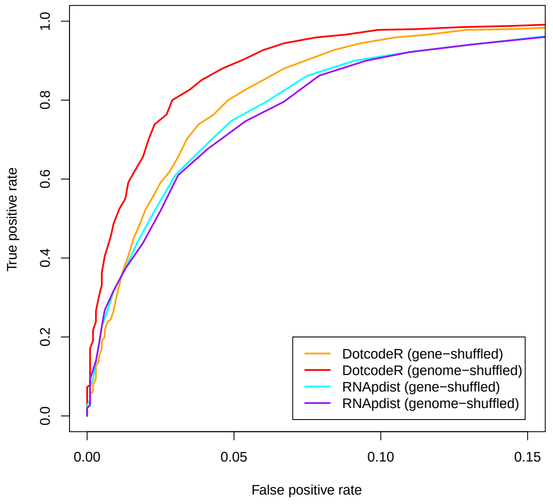

(d) Window size 120, step size 30

Figure S10: ROC curves that show discriminative power of DotcodeR and RNApdist on the test set of simulated short genomes. In this test, we used $d = 1$ for DotcodeR. Note that accuracy was calculated by averaging over all results of the families in the dataset.

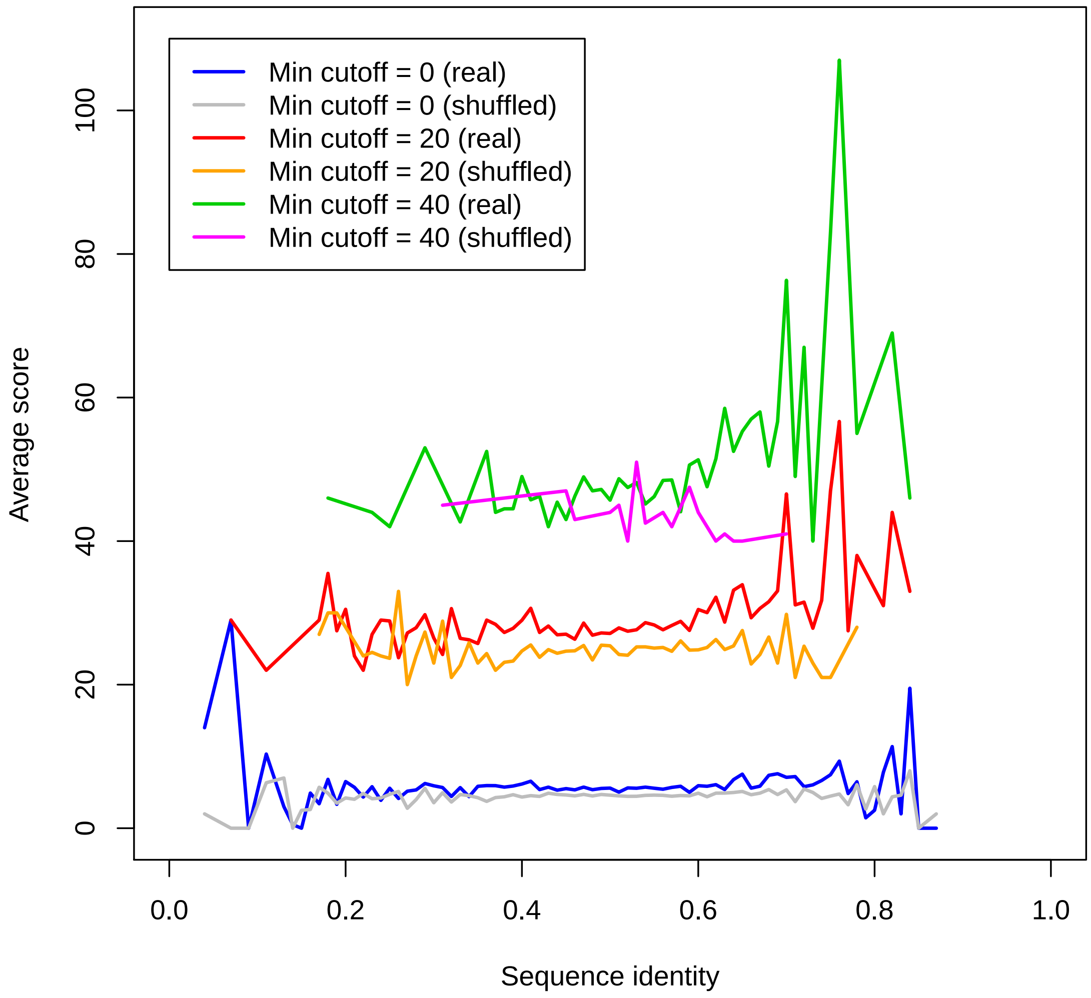

Figure S11: DotcodeR score as a function of sequence identity on the training set of simulated short genomes. The scores used in the y-axis are calculated by averaging over scores in all the families contained in the datasets. The min cutoff $c_{min} \in \{0, 20, 40\}$ means that we consider only scores of at least c_{min} to investigate the relationship.

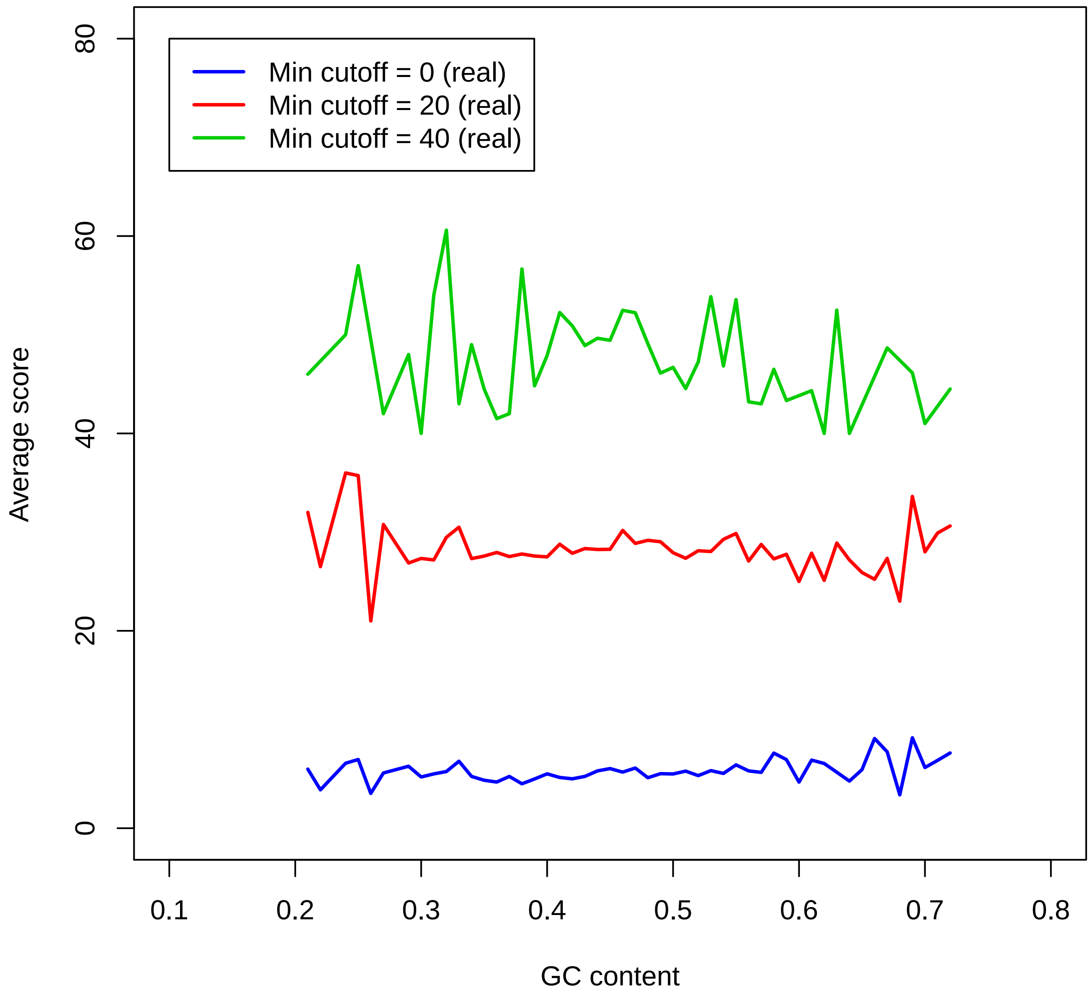

Figure S12: DotcodeR score as a function of GC content on the training set of simulated short genomes. The scores used in the y-axis and the min cutoff can be interpreted in the same way as in Figure S11. Note that GC content was calculated only on real window pairs in the dataset since it should be the same between real and shuffled sequences.

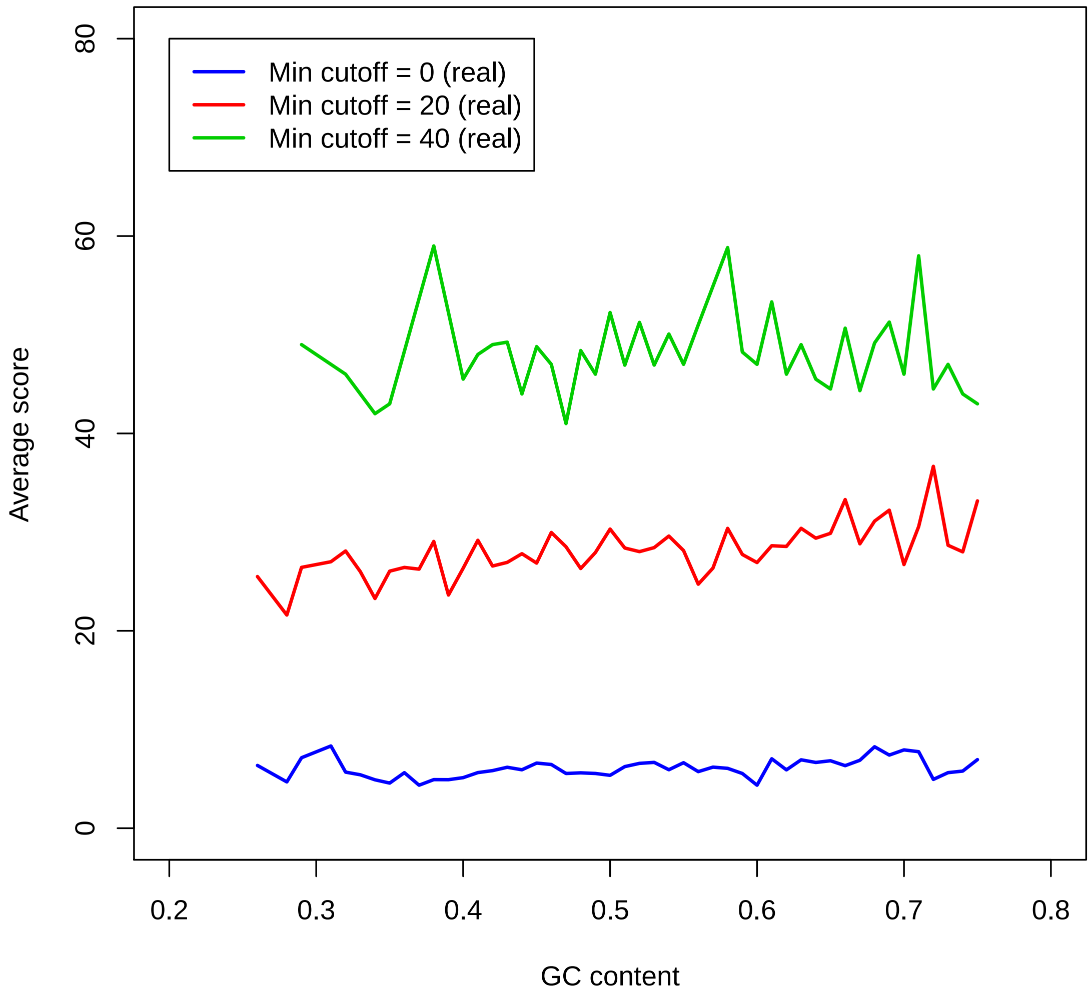

Figure S13: DotcodeR score as a function of GC content on the test set of simulated short genomes. The scores used in the y-axis and the min cutoff can be interpreted in the same way as in Figure S11.

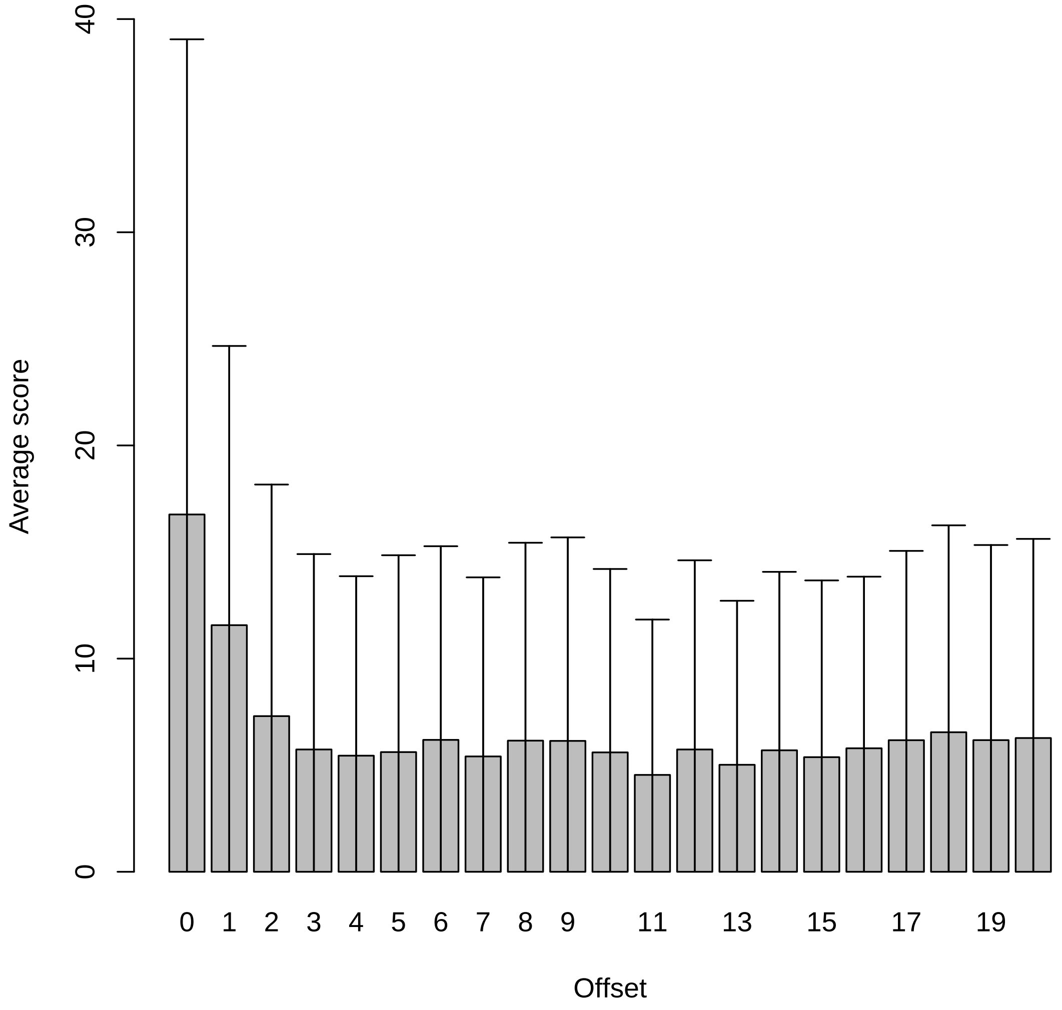

Figure S14: DotcodeR score as a function of window offset between two similar structures. The gray bars show average scores over all real-against-real sequences in the test set, and the error bars indicate standard deviations of the corresponding scores. Note that we used $d = 1$ for DotcodeR.

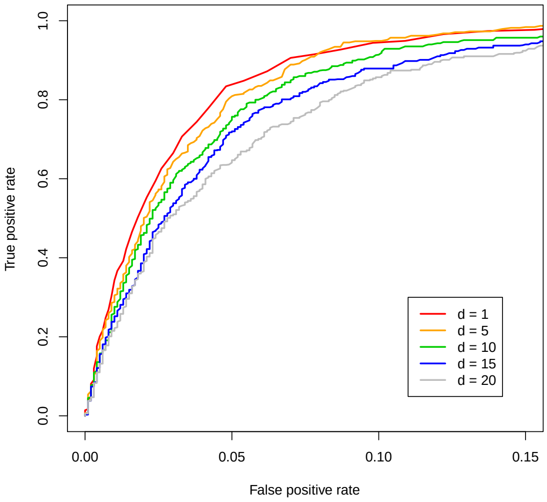

(a) Training set, gene-shuffled

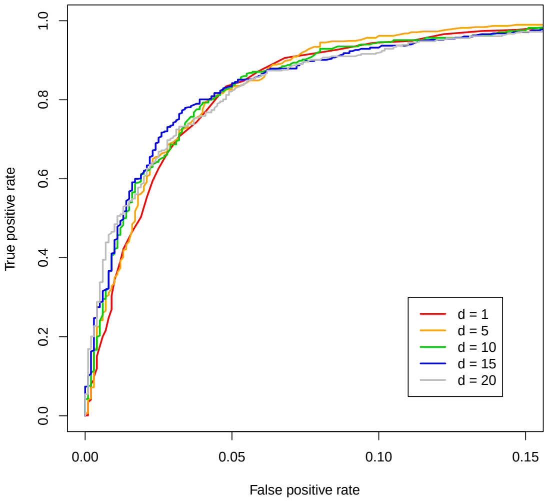

(b) Training set, genome-shuffled

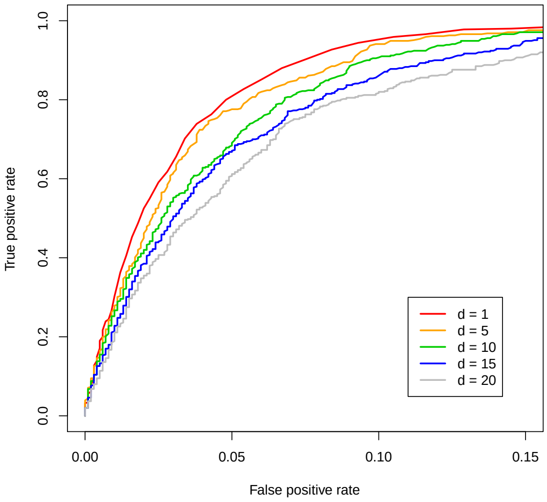

(c) Test set, gene-shuffled

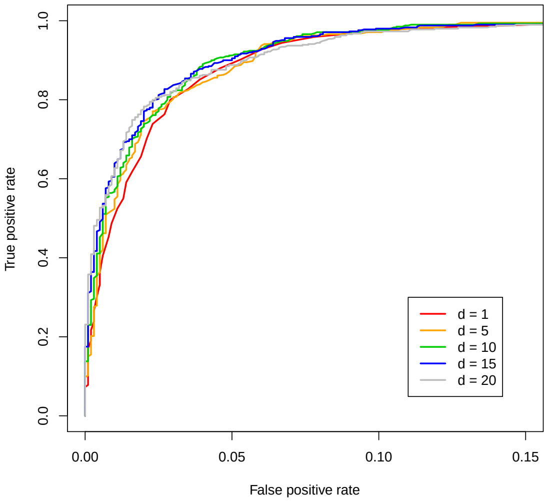

(d) Test set, genome-shuffled

Figure S15: ROC curves for DotcodeR with $d \in \{1, 5, 10, 15, 20\}$ on the benchmark data. In this test, we used the window size of 120 nt and the step size of 30 nt. Note that accuracy was calculated by averaging over all results of the families in the respective datasets.

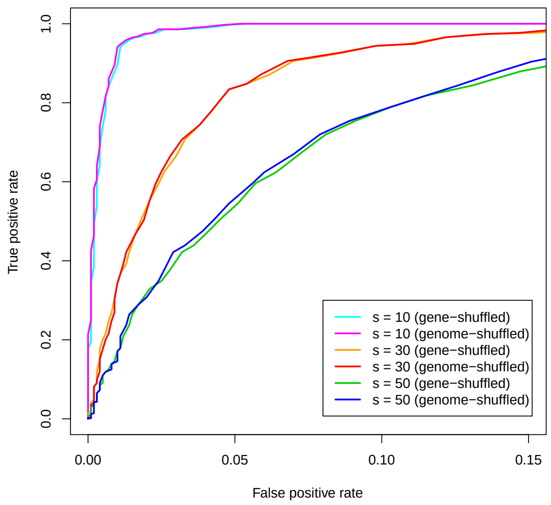

(a) Training set

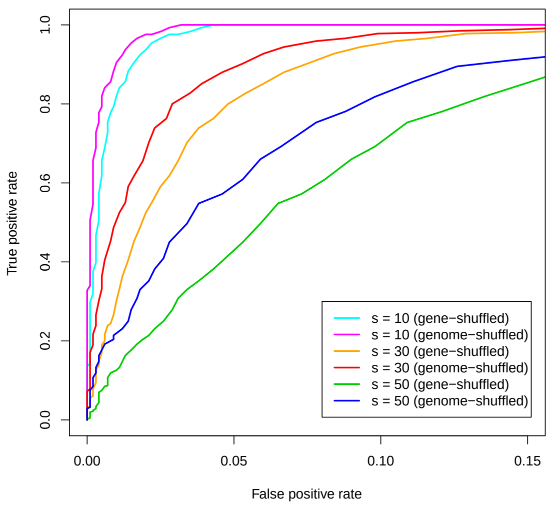

(b) Test set

Figure S16: ROC curves for DotcodeR with the step size $s \in \{10, 30, 50\}$ on the benchmark data. In this test, we used the window size of 120 nt and $d = 1$. Note that accuracy was calculated by averaging over all results of the families in the respective datasets.

S3 Supplementary Notes

S3.1 Parameter settings

We used the Needleman–Wunsch global alignment algorithm to compute similarity scores in DotcodeR with alignment with the following parameters:

- Match score between two binary digits: 9;
- Mismatch penalty between two binary digits: 2;
- Gap penalty: 1;
- Threshold for the sum of the neighboring probabilities in a dot plot: 0.1.

Note that the last parameter was also used in DotcodeR with dot product.

S3.2 Calculating the number of pairs of windows in input

The number of pairs of windows between two chromosomes can be basically calculated by counting the numbers of windows in respective sequences and taking the product of them. In particular, the number of pairs of windows in cleaned input in Table 3 in the main article was calculated as follows:

$$\begin{aligned}
&\#\{\text{pairs of windows in cleaned input}\}\\
=&\#\{\text{pairs of windows in original input}\} - \#\{\text{pairs of windows in repeats}\}\\
&\quad - \#\{\text{pairs of windows in reduced alignments}\}\\
=&1555810 \times 2046609 - 936119 \times 948679 - 280791\\
=&3.184135 \times 10^{12} - 888076436801 - 280791\\
=&2.296058 \times 10^{12},
\end{aligned}$$

where reduced alignments mean the ones obtained by removing pairs of overlapping repeat regions from the original alignments.

S3.3 Estimating run-time for chromosomal screen and genomic screen

The chromosomal screen by DotcodeR on the "original" input took 14.2 CPU months or approximately four days of run-time on a small computer cluster. Taking this and Table 3 in the main article into account, an estimated run-time for the chromosomal screen on the "cleaned" input is

$$\begin{aligned}
&\text{run-time for original input} \times \frac{\#\{\text{pairs of windows in cleaned input}\}}{\#\{\text{pairs of windows in original input}\}}\\
=&14.2 \times \frac{2.296058 \times 10^{12}}{3.184135 \times 10^{12}}\\
=&14.2 \times 0.72\\
=&10.2 \text{ (CPU months)},
\end{aligned}$$

or $4 \times 0.72 = 2.9$ (days) on the small computer cluster.

Next, let us consider the full genomic screen. The number of window comparisons between human and mouse genomes of size 3G bases is estimated as

$$\frac{\{3 \times 10^9 \times (1 - 0.5) - 120\}^2}{30^2} = 2.5 \times 10^{15}$$

due to the theoretical $O(\frac{(L-w)^2}{s^2})$ comparisons described in the main text. Note that approximately 50% of the genomes are assumed to be repeats [1], and thus we remove such regions in the above calculation. An estimated run-time for the full genomic screen is:

$$14.2 \text{ CPU years} \times \frac{2.5 \times 10^{15}}{3.2 \times 10^{12}} = 11100 \text{ CPU months} = 925 \text{ CPU years},$$

which would take:

$$4 \text{ days} \times \frac{11100 \text{ CPU months}}{14.2 \text{ CPU months}} = 3130 \text{ days} = 8.6 \text{ years}.$$

to run on the current cluster.

S3.4 Criterion of determining repeat and aligned regions in annotation

Assume that a known annotated region in a genome (e.g., exon) is overlapped with a known repeat region. Let r_a be a non-overlapping region in the annotation and $|r_a|$ be the length of that region. If $|r_a| < 2s$ where s is a step size of the sliding window, we will judge this annotated region as “repeat.” A known aligned region can be interpreted similarly but in a pairwise way.

References

[1] Treangen, T. J. & Salzberg, S. L. Repetitive DNA and next-generation sequencing: computational challenges and solutions. *Nat. Rev. Genet.* **13**, 36–46 (2011).
